# Hiding the Rabbit: Using a genetic algorithm to investigate shape guidance in visual search

**DOI:** 10.1167/jov.22.1.7

**Published:** 2022-01-13

**Authors:** Avi M. Aizenman, Krista A. Ehinger, Farahnaz A. Wick, Ruggero Micheletto, Jungyeon Park, Lucas Jurgensen, Jeremy M. Wolfe

**Affiliations:** 1 University of California Berkeley, Berkeley, CA, USA; 2 University of Melbourne, Melbourne, Australia; 3 Harvard Medical School, Cambridge, MA, USA; 4 Brigham and Women's Hospital, Cambridge, MA, USA; 5 Yokohama City University, Yokohama, Japan; 6 Harvard University, Cambridge, MA, USA; 7 Cornell University, Ithaca, NY, USA; 8 Harvard Medical School, Cambridge, MA, USA; 9 Brigham and Women's Hospital, Cambridge, MA, USA

**Keywords:** visual search, shape perception, genetic algorithm, attention, evolution

## Abstract

During visual search, attention is guided by specific features, including shape. Our understanding of shape guidance is limited to specific attributes (closures and line terminations) that do not fully explain the richness of preattentive shape processing. We used a novel genetic algorithm method to explore shape space and to stimulate hypotheses about shape guidance. Initially, observers searched for targets among 12 random distractors defined, in radial frequency space, by the amplitude and phase of 10 radial frequencies. Reaction time (RT) was the measure of “fitness.” To evolve toward an easier search task, distractors with faster RTs survived to the next generation, “mated,” and produced offspring (new distractors for the next generation of search). To evolve a harder search, surviving distractors were those yielding longer RTs. Within eight generations of evolution, the method succeeds in producing visual searches either harder or easier than the starting search. In radial frequency space, easy distractors evolve amplitude × frequency spectra that are dissimilar to the target, whereas hard distractors evolve spectra that are more similar to the target. This method also works with naturally shaped targets (e.g., rabbit silhouettes). Interestingly, the most inefficient distractors featured a combination of a body and ear distractors that did not resemble the rabbit (visually or in spectrum). Adding extra ears to these distractors did not impact the search spectrally and instead made it easier to confirm a rabbit, once it was found. In general, these experiments show that shapes that are clearly distinct when attended are similar to each other preattentively.

## Introduction

Our visual system decomposes the light array falling on our retina into separate features such as color, orientation, stereo cues, motion, size, and shape. These features are processed hierarchically by different areas in the brain: with neurons in V1 processing lines and orientations (Hubel & Wiesel, 1968) which are integrated by neurons up the hierarchy where more extended contours are processed (Jeffrey, Wang, & Birch, 2002; Pasupathy & Connor, 1999) to still higher areas that detect and recognize objects (Kourtzi & Kanwisher, 2001). At some point along the path from input to object recognition, there is a bottleneck. We can detect the presence of some basic visual properties like color and size across the entire visual field “in parallel” but, in most cases, we cannot perform a similar global search for a complex object like a rabbit or a teapot. Treisman (1985) argued that some basic features could be processed “preattentively,” whereas object recognition required the “binding” of the features of each object, a process that required selective attention to the object.

**Figure 1.**
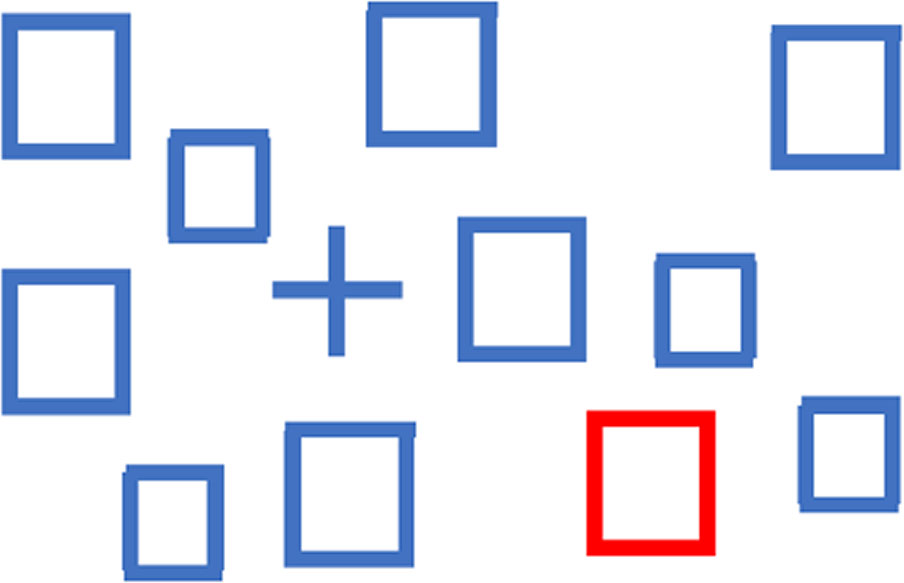
The plus shape and the red square both “pop out” among open blue square distractor items.

Visual search experiments provide a classic line of evidence that a feature is processed preattentively. When a salient feature can be processed preattentively, it will “pop out” in a search, more or less regardless of the number of other, distracting items. Thus in Figure 1 it is intuitively clear that the red item would pop out no matter how many blue items were in the display. Color serves as a basic, preattentive feature, and although there are many complex details (Bauer, Jolicoeur, & Cowan, 1996; D'Zmura, 1991; Nagy & Sanchez, 1990), we can describe the feature space for color quite well.

Returning to Figure 1, it is also clear that the time to search for a plus among boxes would depend very little on the number of open squares in the display (for an account of the small cost of a larger number of distractors, see Ng, Lleras, & Buetti, 2018). Whereas it is clear that the plus and the box differ in shape, defining a feature space for shape is more complex than for attributes like orientation or color. An attribute like orientation can be described by a circular one-dimensional space representing the angle of orientation. Color can be described by any of several three-dimensional spaces. Shape, in contrast, is not so easily described. Indeed, it is unlikely that “shape” is a single feature; it may be more of an umbrella term, covering several attributes.

## Visual search for targets defined by shape

A number of aspects of shape have been identified as supporting highly efficient search in the manner of a simple color or orientation search. These include properties like “closure” (Elder & Zucker, 1993). An open “C” is easy to find among closed “O”s (Treisman, 1985). The target “C” (or plus, above) might also be detected by presence of line terminations (Cheal & Lyon, 1992; Taylor & Badcock, 1988) rather than by the absence of closure. Other shape properties that serve as candidate preattentive features include curvature (Fahle, 1991; Wolfe, Yee, & Friedman-Hill, 1992), curvature discontinuities or corners (Kristjansson & Tse, 2001), Vernier offset (Fahle, 1991), and topological status (Chen, 2005). Beyond that, it is clear that other aspects of shape guide attention (e.g., Cheal & Lyon, 1992; Grunau, Dubé, & Galera, 1994; Pomerantz & Pristach, 1989), but it is not entirely clear what those might be.

**Figure 2.**
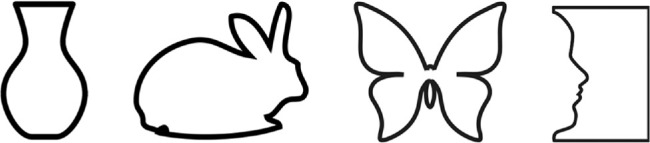
What are the visual primitives describing shape?

Efforts have been made to parameterize the shape feature space, for example, by using radial frequency or RF patterns (Bell, Badcock, Wilson, & Wilkinson, 2007). The RF pattern is a circle whose radius is distorted by amplitude and radial frequency combinations. RF patterns are usually used to represent simple closed-contour shapes but complex shapes approximating those in Figure 2 can be created by combining different radial frequencies in the same way that complex one- or two-dimensional patterns can be produced as the sum of different sinusoidal gratings.

Additional work has taken a parts-based approach, where the visual system represents shapes of complex objects as a hierarchy of simpler parts. A parts-based description allows for a decoupling of the representation of shapes as individual parts from the spatial relationship between these parts. Supporting psychophysical work has shown that the visual system represents shapes in terms of parts (Biederman, 1987; Cave & Kosslyn, 1993). Hoffman & Richards (1984) found an important cue for segmenting a shape into its component parts is the presence of negative minima of curvature along the bounding contour (regions with negative curvature divide a shape into parts). Evidence suggests these part boundaries are computed preattentively (Wolfe & Bennett, 1997).

A complementary approach to parts-based shape representation relies on shape skeleton and axes representations. A shape skeleton is a geometric model based off the medial axis of the shape. For most shapes, the axes are organized in a hierarchical fashion such that a set of parent axes describe the coarse global geometry of the shape, while smaller axes describe individual and smaller component parts. Importantly, methods have been formalized to compare the similarity between shape skeletons, providing a quantitative metric to compare shapes by (Sebastian, Klein & Kimia, 2004; Ayzenberg & Lourenco (2019)). A number of psychophysical results suggest a privileged role for skeleton representations in human object perception (Kimia, 2003; Kovács, Fehér, Julesz, 1998, Siddiqi et al., 2001, Wilder et al., 2011). Neuroimaging studies have additionally shown sensitivity or coding of medial axis or skeleton representations in visual cortical areas (as early as V1 and V4), as well as areas known to contribute to object recognition such as IT (Lee, Mumford, Romero, & Lamme, 1998; Pasupathy & Connor, 2002; Hung, Carlson & Connor, 2012).

Despite the many representations of shape available, our understanding of the role of shape in visual search is limited in part because it is not clear that any one parameterization covers all shapes. Without a general framework to describe shape, how can we discover basic principles that might explain why one closed-curve shape is easy to find among others? Prior visual search work has tended to rely on the intuitions of the experimenters to parameterize shape, and has focused on easily-defined aspects of simple shapes, such as line terminations and curvature. However, some aspects of shape may not be so easily defined, so this approach is unlikely to give a complete picture of how target shape guides attention in visual search. Instead of starting from predefined shape features, an alternative approach might be to run a large-scale search experiment with random shape targets and distractors and to explore the resulting search data to try to automatically discover what features make a search task easy or difficult. However, this would be a massive undertaking, and only a subset of the random target-distractor pairings would likely give interesting results (see Huang, 2020 for one effort). In this work, we follow the feature discovery approach, but we use an optimization algorithm as a technique to more efficiently search this space. Specifically, we use a genetic algorithm (GA) method (Holland, 1992) in an effort to automatically discover shape features that guide visual search. To anticipate our results, the GA method succeeds in generating easier and harder searches in an automatic manner. It does not immediately clarify the basis of visual search for shape, although it does support the roles of similarity (Duncan and Humphreys, 1989) and of part-based models of shape, described above.

## Genetic algorithms

GAs belong to a class of stochastic search optimization techniques that are inspired by natural selection, the biological process that drives evolution. They exploit a fitness function to direct search to a region of better (or worse) performance. A GA typically starts with randomly sampled states. Successor states are generated by combining two parent states rather than modifying a single state. A fitness parameter or score guides how the population of states evolves; which items have “offspring.” From one “generation” to the next, states that are poor fits for the problem die out, whereas the better states are selected and accepted to continue to the next generation, in a survival of the fittest solution. These offspring states acquire some “genes” (or parameters) from one parent and some from the other, with some small probability of “mutation” included to allow for a heterogeneous population. Depending on the parameters of the GA, the result of many such generations is a final state or small population of final states with high fitness. A key advantage of GA compared to other stochastic optimization techniques is that it makes very few assumptions about how the state space is organized or how the “gene” parameters are related to the fitness function.

In general, GAs have been used to solve complex problems across different scientific fields such as modeling global temperature changes, optimizing the selection of financial portfolios, and improving the abbreviation of psychological questionnaires (Sahdra, Ciarrochi, Parker, & Scrucca, 2016; Sefiane & Benbouziane, 2012; Stanislawska, Krawiec, & Kundzewicz, 2012). GAs have also been used to manipulate the difficulty of search displays (Van der Burg, Cass, Theeuwes, & Alais, 2015). In their experiment, observers started with a set of random search displays made up of distractors comprised of three orientations (0°, 10°, or 90°) and three colors (red, green, or blue) and searched for a target that was a red horizontal line. After completing this first set of trials (or the first generation), the displays that led to the fastest reaction times (RTs) were selected. The distractors from these displays were allowed to “mate,” exchanging features to produce a new set of “offspring” distractors to be used in the next set (or generation) of search trials. This process of selecting the fastest search displays and allowing the distractor features to propagate over the subsequent generations led to a decrease in average RT over the course of multiple generations. Over time, the displays contained fewer red 10° targets, thus making search for the red horizontal target easier. Interestingly, and perhaps counterintuitively, the number of green and blue horizontal lines increased over generations. One would expect that these blue and green horizontal lines should have impeded visual search, because these distractors share a feature (orientation) with the target line. However, this configuration led to faster search. In the GA approach, the observers’ own behavior is used to select the visual features that makes search easy or hard. Van der Burg and colleagues’ findings show that in conjunction search for color and orientation, observers restrict their search to items with the same color as the target, as opposed to items with the same orientation as the target. This suggests that the presence of same-color distractors in the display is the greatest limitation on search performance.

We use a similar approach to explore the feature space of closed contours. Instead of an experimenter selecting a small and potentially biased handful of shape exemplars, an observers’ own performance as measured by RT can guide exploration of combinations of RF patterns that make search easy or difficult. By comparing reaction time over generations, as well as the “genome” of the distractors across generations, we can “evolve” search displays based on observers’ behaviors and at the same time quantify which local attributes are propagated over generations.

In the context of our shape search problem, the states are RF patterns, each with a unique combination of radial amplitude and phase parameters. Initially, we create distractors by randomly sampling this space; each set of 12 RF pattern distractors is called a population. Observers search for a target shape (which is also an RF pattern) among each distractor in the population and RTs are collected. The next generation of RF patterns is produced by selecting the displays with the highest or lowest reaction times. Pairs of RF patterns “mate” by randomly recombining their amplitude and phase parameters on each radial frequency. Occasionally, these parameters may randomly “mutate” to maintain diversity in the distractor population.

We used RT as our fitness function to evaluate performance and to select displays for evolution, but other measurements could have been used in the fitness function. For instance, we could have used a more complex fitness function involving RT and eye movements, but that would have not only made the evaluation score more complex to interpret but also may have required more experimental trials to converge to the optimal solution. In any case, the use of RT as the fitness function in this experiment and the use of RF as the basis function should be seen as one way to apply GA methods to the problem of shape search; not the only way. As will be seen in the results, these choices produce some interesting results, but they do not “solve” the problem of visual search for shape. Other aspects of that solution may be uncovered by using other basis functions to create stimuli and other fitness functions to measure responses to those stimuli.

In separate blocks, we have used the longest RTs or the shortest RTs to define “fitness.” If, as in Van de Burg et al. (2015), it is the fastest search displays that survive in this “survival of the fittest” GA implementation, then displays can evolve over time to produce significantly faster, easier searches. However, one can also define the slowest search displays as “fittest.” In this case, the next generation of distractors is selected from the *slowest* and most challenging search displays. Propagating these shapes as distractors should lead to slow and demanding searches. In the former case, we may identify shape features that distinguish targets and distractors. In the latter case, we may identify those distractor features that hide the target from immediate detection. We approached parameterizing the target and distractor shapes using radial frequency and skeleton representations, as well as a method of quantifying total curvature (perimeter^2^/area). By looking at target-distractor differences over the course of evolution, we can hope to determine whether some shape parameters are more effective than others at revealing the shape features that control search.

## Experiment 1: The basic genetic algorithm method

In Experiment 1, we use a genetic algorithm (Whitley, 1994) to modify the shapes of distractor items in a search display to make search for a target shape easier or harder. On each trial, observers search for the target shape in an array of homogeneous distractor shapes. At the start of the experiment, these distractors are randomly generated according to rules described below. Each of the distractor types is used during a block of search trials in which the RT required to find the target is measured. At the end of the block, the distractors are ranked in order of the RTs they produced. Based on the RTs, the distractors producing the longest (for evolve hard) or shortest (for evolve easy) search times are used to produce a new “generation” of distractor types. The distractors that survive are allowed to “mate” and to produce new distractors. The mating process shuffles the parental “genes” and is subject to some rate of mutation. This process is repeated over multiple generations in order to create new populations of distractor shapes that result in either slower or faster search for the target shape.

Since the evolution is driven only by search times, there is no constraint on what shape features the genetic algorithm can learn to make the search harder or easier. It is also not guaranteed that the final generation distractors for a particular target shape will all be similar to each other, either within or across observers. Analyzing the distribution of evolved “hard” and “easy” distractor shapes may yield some insights into what kinds of features guide search for shape, since we expect the genetic algorithm process to enhance these features when evolving harder distractors and suppress these features when evolving easy ones.

### Methods

Data were collected from 12 participants (eight females, four males, mean age 26.5 years). All observers had normal or corrected to normal vision and passed the Ishihara color test. All experimental procedures were approved by the Brigham and Women's Hospital Institutional Review Board, and all participants gave informed consent and were paid $10 an hour. The number of observers was chosen based on prior search experiments of this sort. This experiment is essentially exploratory, meaning that we cannot estimate effect sizes. However, over many years of visual search experiments, we can estimate that the standard deviation of the slopes of an RT × set size function for a given task will be about 0.3 of the slope. For RTs, the standard deviation is about 0.4 of the RT. Detecting a 1.5× change in slope, for example, with *p* = 0.05 and power = 0.95 requires 11 observers.

### Apparatus and stimuli

Experimental sessions were carried out on a 24″ iMac 2009 computer (model A1225) with a resolution of 1920 × 1200 pixels, with a 60 Hz refresh rate. Experiments were written in Matlab 7.10 (The MathWorks) using the Psychophysics Toolbox (Brainard, 1997; Pelli, 1997). Observers were placed so that their eyes were 57 cm from the monitor. At this viewing distance, 1 cm subtends 1° of visual angle (°).

**Figure 3.**
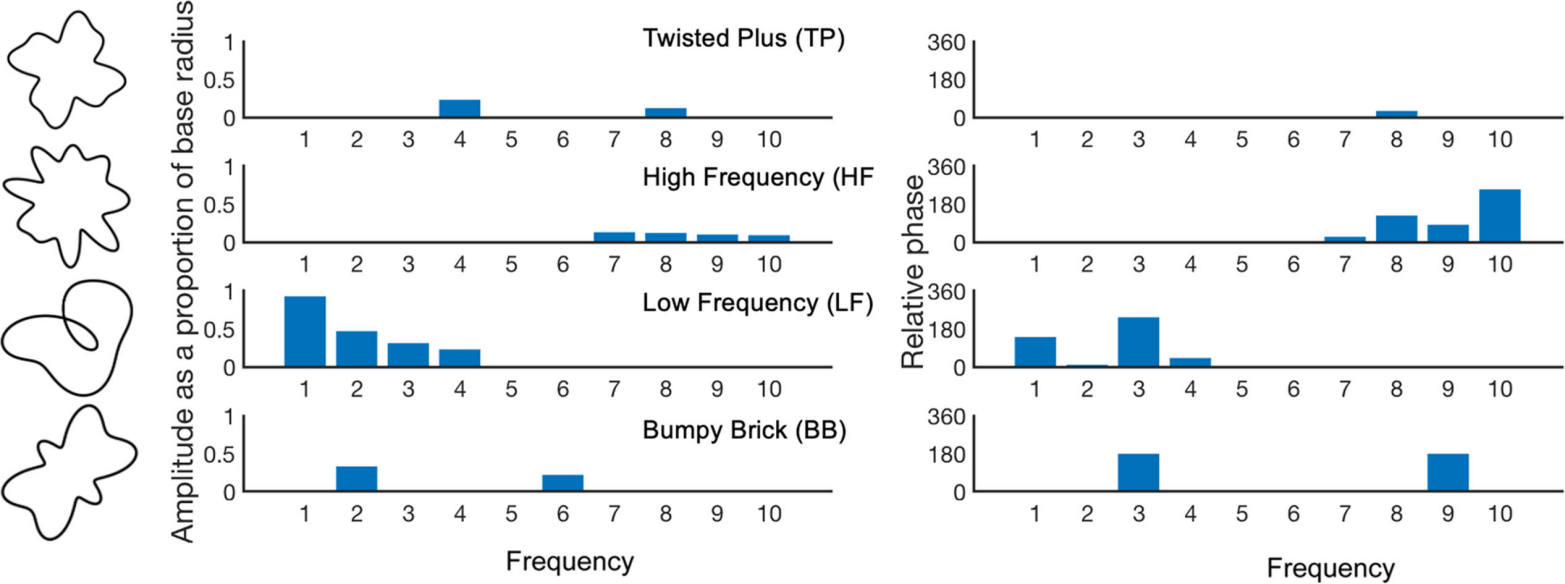
Examples of the four target types. Shown next to each target is their associated amplitude spectra and relative phase. Frequencies (1–10) are shown on each of the x axis, amplitude as a proportion of radius is shown on one y axis, relative phase on the other. The abbreviations associated with each target type are additionally shown.

In each trial participants were shown 16 or 24 items in a circular array on the screen and a target was always present. In every trial, one item was the target and all other items shown were homogenous distractors rotated to different orientations. Participants were asked to find a target amongst the distractors and to click on the target as quickly as possible. There was a blue fixation target at the center of the array, and at the start of every trial the mouse cursor was relocated to the center of the display. There were four different types of targets, each tested separately. As shown in Figure 3, these were a high frequency (HF) target, a low frequency (LF) target, a bumpy brick (BB) target, and a twisted plus (TP) target. Figure 3 also shows each target's amplitude spectrum. To reduce the effects of low-level features like luminance (which might differ across filled shapes because of differences in area), targets and distractors were displayed as green outline contours (RGB = 83, 187, 121) on a black background.

**Figure 4.**
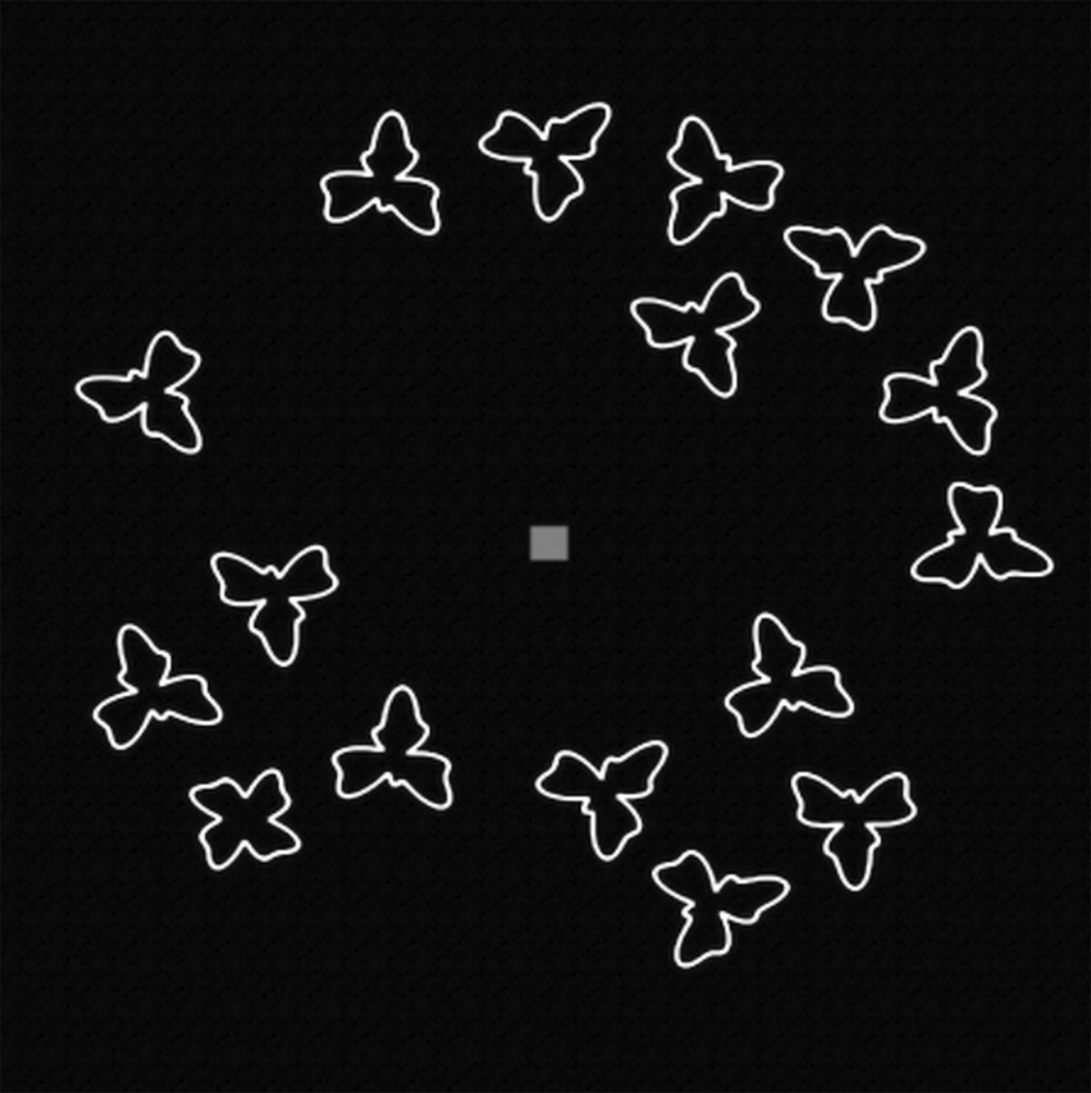
Examples of the search array for set size of 16 items. Note the color of the targets has been changed from green (used in the experiment) to white for the sake of visibility. The target is the “twisted plus” item with four lobes. Note that it is not particularly easy to find, although it is easy enough to identify, once attended.

The experiment consisted of eight blocks: four target types by Evolve-Easy or Evolve-Hard evolution. Within each of the experimental blocks there were eight “generations” of 24 trials each. Within a “generation,” each of the 12 distractors was tested twice in a generation, once in a set size of 16 and once in a set size of 24 in a homogeneous search, meaning each display contained identical distractor items (an example display is shown in Figure 4). The set sizes were run sequentially (12 trials at set size 16, followed by 12 trials at set size 24) with the order of distractors randomized within each set size. After each generation of 24 trials, the distractor pool “evolved” based on the distractors that had led to the fastest search (Evolve-Easy) or the slowest search (Evolve-Hard).

The target and distractor shapes were radial frequency patterns: sums of sinusoidal variations of the outline of a circle with a radius of 30 pixels. Only the first 10 spatial frequencies were used to build the shapes. Higher frequencies have zero amplitude. Each target had non-zero amplitude on two to four frequencies; the amplitudes and relative phases of the target shapes are given in Figure 3.

The distractor shapes were defined by their 10 amplitudes and phases, plus an additional 10 binary values which could turn a given frequency “on” or “off” by setting its amplitude to zero. This binary vector was included to help make the distractor shape distribution more similar to the target shapes, which had zero amplitude on most frequencies. For the first generation of each experiment block, an initial set of 12 distractor shapes were created by assigning random amplitudes and phases to each of the 10 frequencies. Amplitude was chosen from a uniform distribution randomly from the range 50–150, and was then divided by the frequency value (thus, frequency 2's initial amplitude range is 25–75, frequency 3's initial amplitude range is 16.7–50, etc.) This approximates the 1/f rule for the Fourier spectra of real scenes (Black, Hurst, & Simaika, 1965). Phase was randomly selected from a uniform distribution from 1-360 degrees.

**Figure 5.**
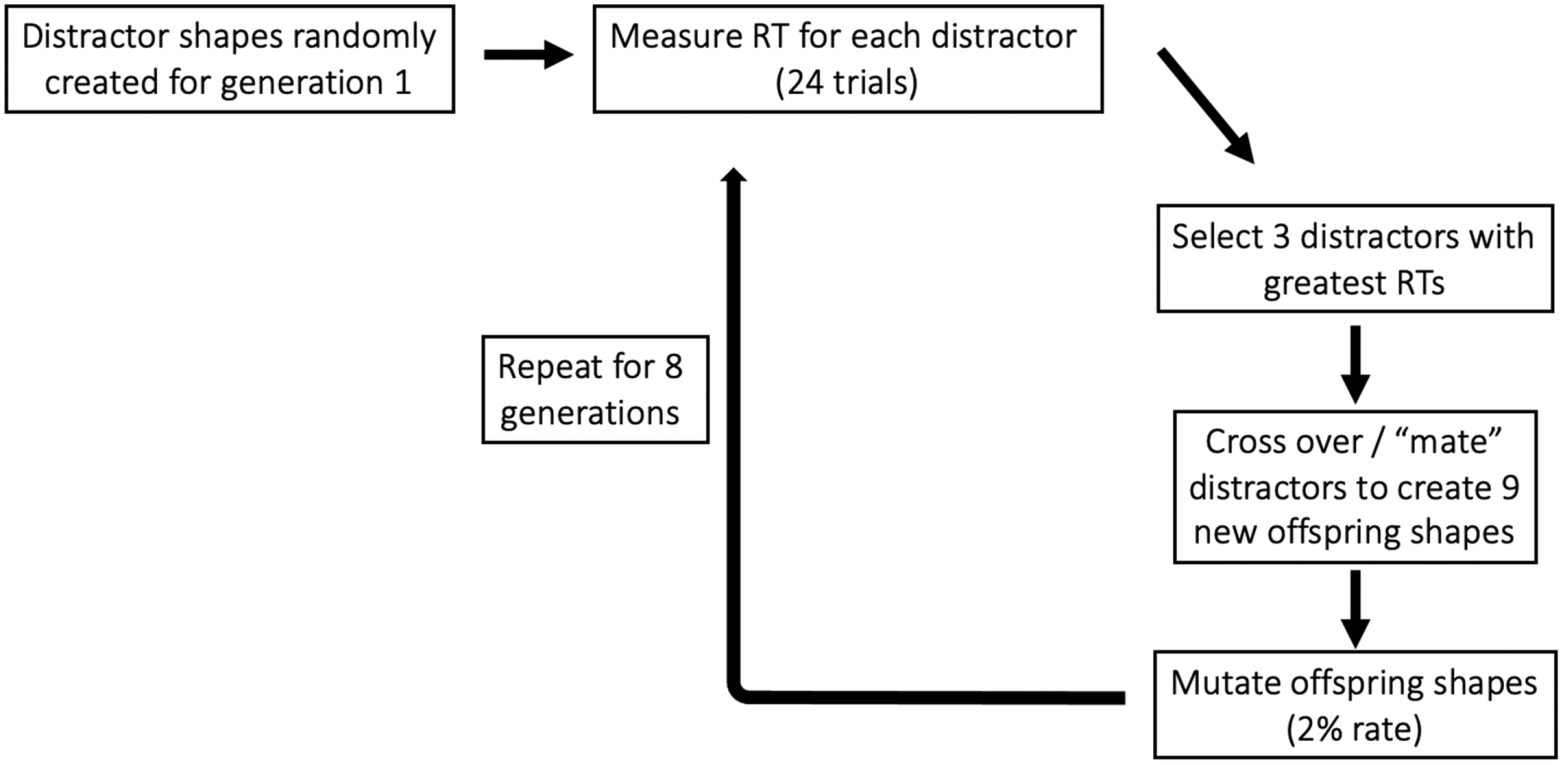
Schematic for the “Evolve-Hard” evolution process in selecting the fittest distractors to propagate to the next generation. The “Evolve-Easy” process was identical except that the distractors with the lowest mean RT were selected and crossed over.

The initial sets of distractors were randomly generated, but distractors on subsequent generations were created using the genetic algorithm. On blocks where the evolution type was set to Evolve-Easy, the three distractors (or “parents”) that produced the fastest search times (mean RT) were selected to create the next generation of distractors. This new pool of 12 distractors consisted of the three parents plus nine “offspring” generated by randomly mixing parents’ “genomes,” which are 30-element vectors consisting of amplitudes, phases, and on/off binary values for each of the 10 frequencies that defined the radial basis pattern shapes. An offspring genome was created by randomly selecting two parent genomes and crossing them over, so that each element in the offspring genome has a 50% probability of coming from either parent. The formula for the amplitude and phase of a given frequency as a function of the two parent genomes can be found in Appendix A. A mutation rate of .02 was included so that each of the 30 elements had a 2% chance of undergoing a random change. Changes included randomly turning a frequency on or off (i.e., changing the binary value from 0 to 1, or 1 to 0), increasing or decreasing amplitude by a factor of 2, or changing phase to a uniform random value between 1° and 360°. This mutation rate allows for some noise in the evolution process to help prevent the algorithm from evolving toward a local minimum. A similar process was used in the Evolve-Hard condition, except the three trials that had led to the *slowest* search times were selected and crossed over. Instead of averaging the response times for each distractor as in the Evolve-Easy condition, we used a weighted sum of the mean RTs and slope of the RT × set size function (slope − 0.15 × mean RT), as the RT × set size function is the standard measure of search difficulty. This process was used to promote distractors to produce a more difficult search. A schematic for this process is shown in Figure 5. The new “evolved” distractors were then presented in the next generation after which the same evolution process occurred. This cycle continued for eight generations of 24 trials each.

Observers participated in one experimental session. Each observer was presented with a block of eight generations of 24 trials each for each of the four stimulus types shown in Figure 3 with separate blocks of Evolve-Easy trials and Evolve-Hard trials. Order of target type and evolution type (easy or hard) was counterbalanced across observers.

### Results

**Figure 6.**
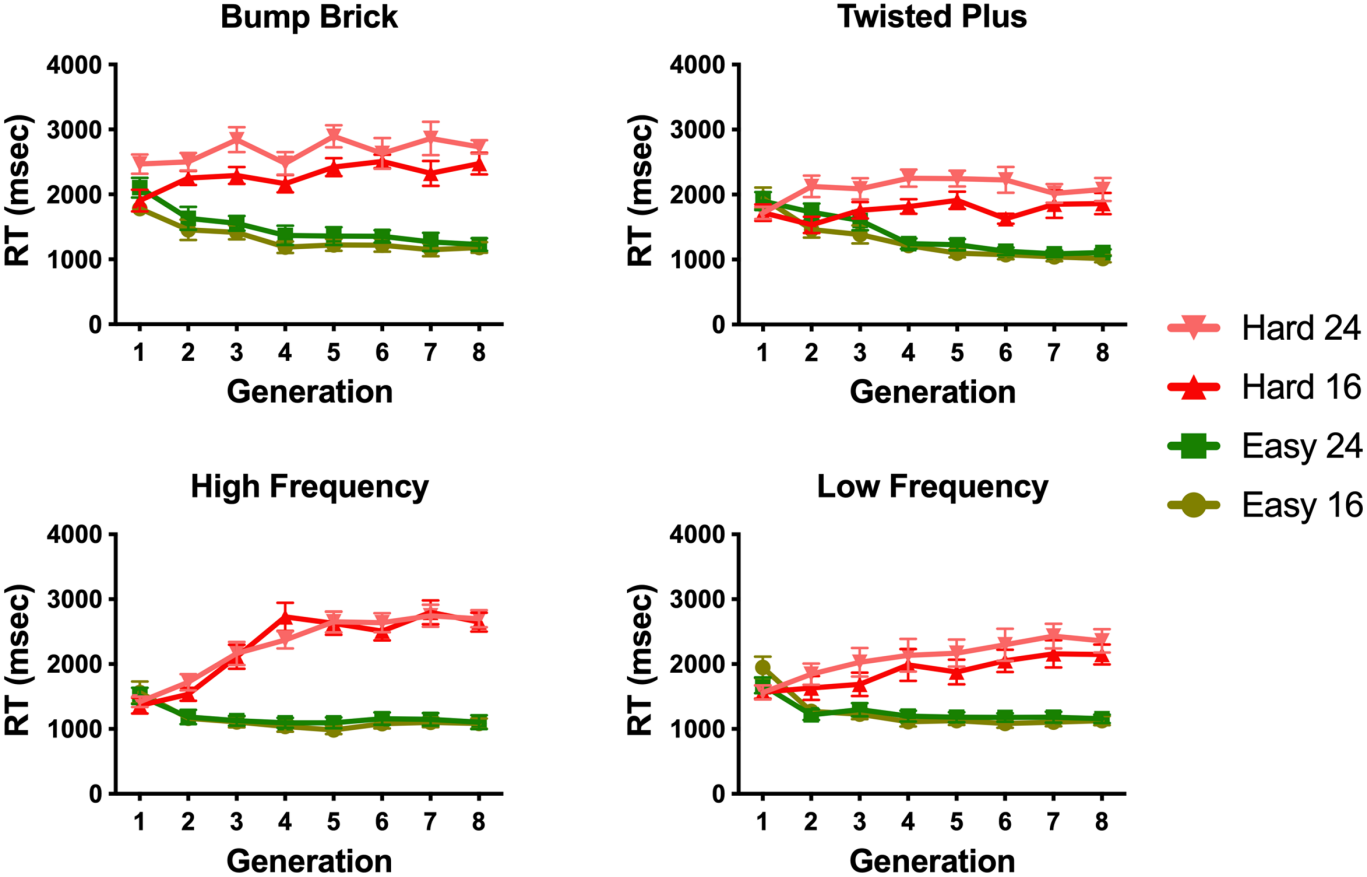
Average RT in msec by generation. Green lines correspond to the evolution direction set to increase the efficiency of the search (Evolve-Easy), while red corresponds to increasing the difficulty of the search (Evolve-Hard). Error bars in all plots correspond to standard error of the mean.

If the GA method for distractor evolution works, distractor shapes should evolve over time to increase or decrease the difficulty of search. Figure 6 shows the average RT as a function of generation for eight generations in each evolution condition and for each set size. It is clear that the RTs in the Evolve-Easy and Evolve-Hard conditions start in the same place in generation 1 and then diverge under the different evolutionary pressures. In the Evolve-Easy conditions, RTs become shorter. In the Evolve-Hard conditions, they become longer. For each of the four target types. A 2 (evolution condition) × 4 (target type) repeated-measures analysis of variance (ANOVA) on the mean RTs from the first generation shows a main effect of target type (*F*(3,33) = 18.65, *p* < 0.001, $\eta_{G}^{2}$ = 0.63). Some targets are intrinsically easier to find than others. There was no significant effect of evolution direction on first generation RTs (*F*(3,33) = 2.51, *p* = 0.14), and the interaction between target type and evolution direction was significant (*F*(3,33) = 3.45, *p* = 0.03, $\eta_{G}^{2}$ = .24). In the first generation, Evolve-Hard and Evolve-Easy distractors are all randomly generated, so we expect the search times in the Evolve-Hard and Evolve-Easy conditions to be similar. However, the baseline difficulty of the search task varies across the four target shapes. Any comparison quantifying differences in RT as a function of evolution between target types must take this into consideration.

**Figure 7.**
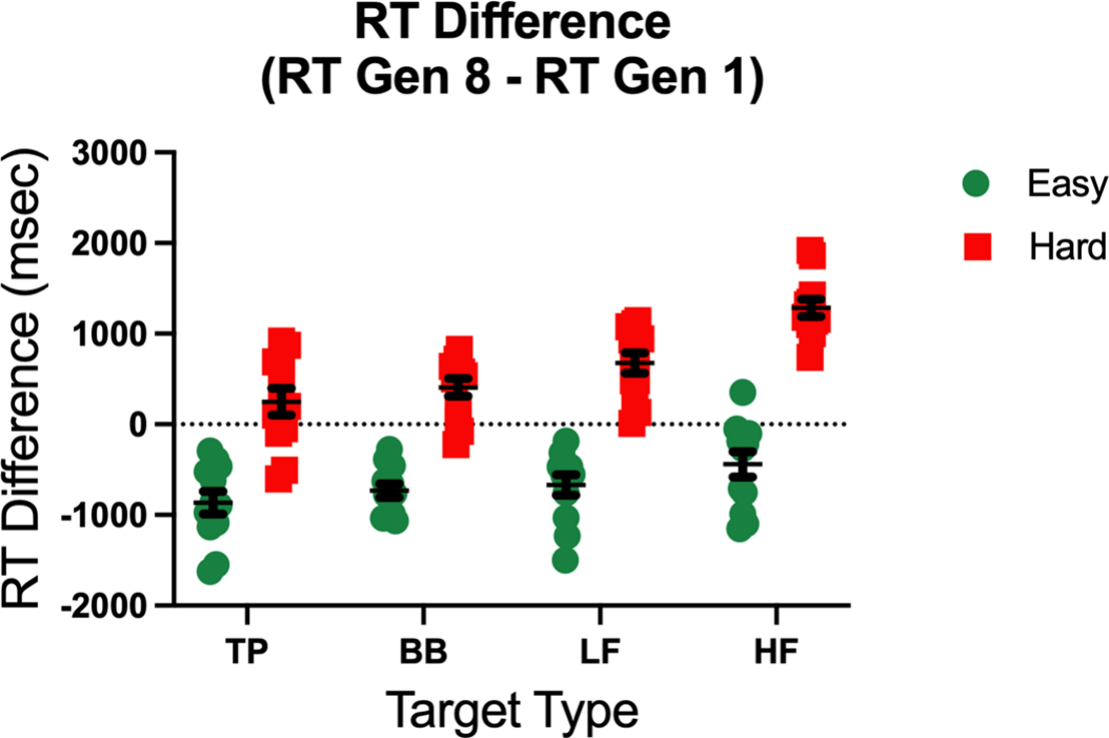
The average difference in RT between generation one and generation eight for all conditions. Red and green points correspond to individual observers in the Evolve-Hard and Evolve-Easy condition, respectively. The dotted line represents an RT difference of 0, or no change in RT over eight generations. Data points would be expected to fall on this line if there was no evolution over generations. Error bars correspond to standard error of the mean.

Figure 7 shows the change in average RT between the first and last generation for each target and evolution direction for each observer. On average, RTs in the Evolve-Hard condition increased between the first and last generation, while RTs in the Evolve-Easy condition decreased. A 2 (evolution condition) x 4 (target type) repeated measures ANOVA of these RT differences shows a main effect of evolution direction (*F*(1,11) = 163.99, *p* < 0.001,$\eta_{G}^{2}$ = .94) which suggests that the GA method achieves the goal of producing harder distractors in the Evolve-Hard condition and easier distractors in the Evolve-Easy condition. There is also a main effect of target type (*F*(3,33) = 26.73, *p* < 0.001,$\eta_{G}^{2}$ = .71), but no significant interaction (*F*(3,33) = 2.47, *p* < 0.08), which may reflect how close the initial generation's average RTs were to ceiling or floor performance.

**Figure 8.**
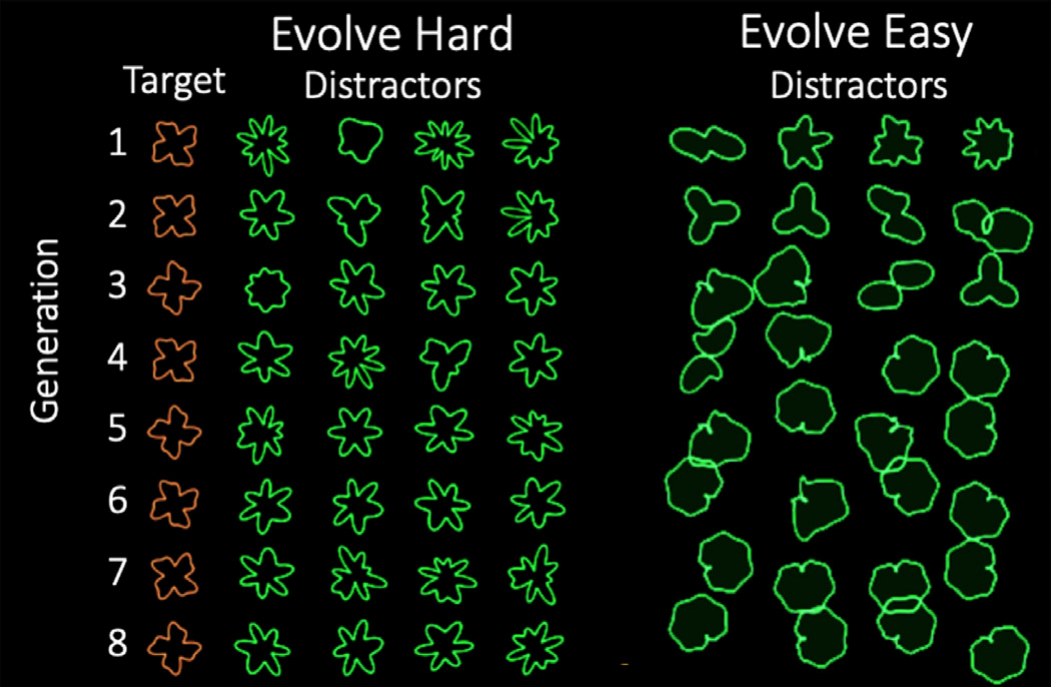
Sample Evolve-Easy and Evolve-Hard evolutionary trajectories in the search for a “twisted plus” target.

The next question is how the features of the distractor shapes change with evolution, and how this corresponds to changes in RT over generations. Figure 8 shows examples of Evolve-Hard and Evolve-Easy evolution for the TP target for one, illustrative observer. The evolutionary paths of four distractors are shown. The result seems intuitively reasonable, but note that the “hard” distractors would not be mistaken for the target. They are quite different shapes, but they are shapes that hide the target (as in Figure 4). We quantified the similarity between target and distractor shapes by using several shape parameterization methods including skeleton representations, perimeter^2^/area (a measure of total curvature), and radial frequency representations. If any one of these metrics provides a meaningful measure of human shape similarity, the target distractor difference of the metric should change over generations in a way that is consistent with the direction of RT changes − RT should increase or decrease with target-distractor similarity.

To compute the skeleton representation for the targets and distractors tested, we used the *bwmorph* function in MATLAB, which generates skeletons derived from morphological thinning. The number of iterations was set to infinity. We quantified the similarity between the shape skeletons of target and distractor shapes for each generation using the skeleton similarity metric from Ayzenberg and Lourenco (2019). Briefly, this method calculates the mean Euclidean distance between each point on the distractor skeleton and the closest point on the target skeleton following maximal alignment. In order to measure the total curvature of targets and distractors, we computed perimeter^2^/area for each target and distractor. We also measured the similarity between distractors and the target as the distance (L2 norm) between their radial Fourier amplitude spectra.

**Figure 9.**
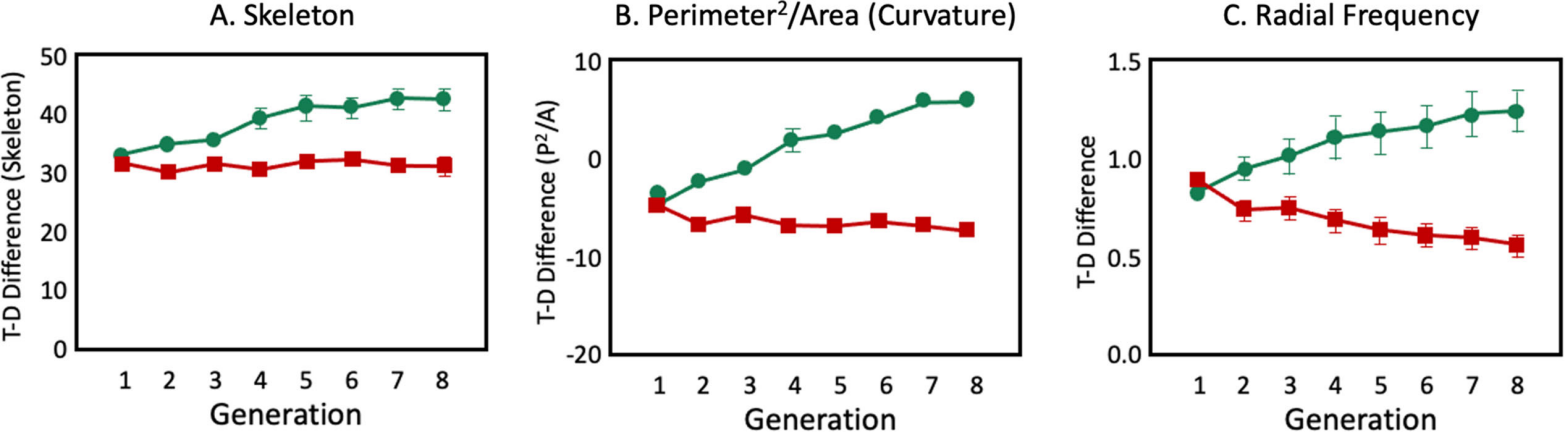
Target distractor similarity as measured using different shape parameterization methods including (A) a skeleton representation, (B) The Target-distractor difference of total curvature as quantified by the perimeter^2^/area metric and (C) Target-distractor difference of the shapes’ radial Fourier amplitude spectra.

Figure 9 shows the results of these analyses for the *Twisted-Plus* target. Across these different shape parametrization techniques we found that in general in the Evolve-Hard conditions, distractors tend to become more similar to the target and in the Evolve-Easy conditions, distractors tend to become less similar to the target. However, the LF target shows the opposite result (please see appendix B for details on these analyses). If more generations were included, instead of limiting the genetic algorithm to 8 generations, would we see a more robust evolution? Appendix C shows a follow-up study that tests whether adding more generations (up to 20) changes these findings. The results suggest that our initial decision of including eight generations is appropriate, because the majority of the evolution occurs in earlier generations.

### Discussion

The results from Experiment 1 show that a genetic algorithm can be used to manipulate the difficulty of a visual search task for a target defined by shape. This method evolves distractors over a series of generations by using observers’ RTs as a measure of fitness, and recombining elements from the most successful distractors to produce harder (or easier) distractor shapes. The changes in RT over generations seem to reflect changes in the similarity between the distractors and the target. The change in similarity can be seen in each of the shape parameterization techniques tested (shape skeletons, total curvature, and Fourier amplitude spectrum). When the algorithm is tasked with making the search more difficult, it generally produces distractors more similar to the target, and when the goal is to make the search easier, it produces distractors less similar to the target.

Results, like those shown in Figure 8 give some insight into the preattentive representation of shape. The TP target has several properties that could be candidate as features. For instance, it is roughly symmetric and has four main lobes. However, the algorithm does not seem to create symmetrical distractors or distractors with exactly four main parts. Instead, the distractors that mask the presence of the target are shapes with multiple lobes or parts of similar size. The number of those parts seem relatively unimportant even though, once we attend to one of these distractors, it is clear that it cannot be a “twisted plus.” When evolving in the easy search direction, the algorithm seems to be eliminating these parts or making them less pronounced.

As shown in Appendix B, the LF target seems to be a somewhat different story. Note that the LF target has a unique feature not found in the other targets—an internal loop that occurs when the radii over some part of the circle are negative. Visual inspection of the final generation of Evolve-Hard and Evolve-Easy distractors for this target suggests that the presence or absence of a loop was an important feature which the genetic algorithm sought to reproduce in the distractor set. The hard distractors for the LF target usually have internal loops, and the easy distractors usually do not have internal loops. The loop feature is not particularly well represented in the Fourier amplitude spectrum of a shape: a very high amplitude on any frequency, or any combination of frequencies, can produce internal loops in these RF shapes. The significance of loops would be consistent with Chen's emphasis on the role of topological status in search (Chen, 2005). The items with loops would be categorically topologically different from items without loops.

## Experiment 2: Are radial frequencies basic features?

In Experiment 1, our genetic algorithm evolved distractor shapes to increase or decrease the difficult of search for a target shape by making the Fourier amplitude spectra of the distractor shapes more similar (Evolve-Hard) or less similar (Evolve-Easy) to the target's amplitude spectra. Perhaps observers were, in effect, searching for specific radial frequencies. Spatial frequencies can serve as basic features in search (Davis, Kramer, & Graham, 1983), so perhaps observers can search for a specific radial frequency in the same way. Indeed, if simple, single frequency stimuli were used, we would expect some ability to guide search based on the number of “bumps” on the outline of the shape (Reijnen, Wolfe, & Krummenacher, 2013). In Experiment 2, we use the genetic algorithm approach to investigate whether the mix of radial frequencies in the target shape act as guiding features for visual search.

There is some evidence that radial frequency patterns are processed by dedicated channels in the early visual system, with separate channels for each of the first few frequencies which may appear in as early as area V2 (Bell, Wilkinson, Wilson, Loffler, & Badcock, 2009; Loffler, 2008; Salmela, Henriksson, & Vanni, 2016). This suggests that the presence or absence of particular frequencies in a shape could be processed quite early in parallel across the visual field and allow a target with a unique radial frequency to “pop out” among distractors which do not contain that frequency. Alternatively, in a shape search task, as opposed to a bump counting task, the presence or absence of a few specific frequencies may not be sufficient to guide attention to a target.

To address this question, we repeated the search task with the four simple shape targets from Experiment 1 and used the genetic algorithm to evolve distractor shapes that made the target harder or easier to find. Unlike the prior experiments, the algorithm was constrained to include the target's radial frequencies in the easy distractors, and it was not allowed to include these frequencies in the hard distractors. If the target's radial frequencies are, in fact, the guiding features in the shape search task, this would cripple the evolution of both easy and hard search. If shape search is based on higher-order shape properties (e.g., frequency, size, and number of parts on a shape), then the algorithm should still be able to evolve hard and easy distractors, perhaps by evolving frequencies near the target frequencies.

### Methods

Data were collected from 12 new participants (eight females, four males, mean age 25.9 years). All observers had normal or corrected-to-normal vision and passed the Ishihara test for color vision. All experimental procedures were approved by the Brigham and Women's Hospital Institutional Review Board, and all participants gave informed consent and were paid $10 an hour.

### Apparatus and stimuli

Experiment 2 was identical to Experiment 1, except that some elements of the distractor genomes were set to fixed values in the initial generation and could not be modified in any subsequent generations. In the Evolve-Easy blocks, the frequencies which were non-zero in the target shape (e.g., two and six for the BB target) were always “on” in the distractor genomes and always had the same amplitudes as the target shape (the phases, however, were initialized to random values and could be changed over generations). In the Evolve-Hard blocks, the frequencies which were non-zero in the target shape were always “off” in the distractor shapes and had zero amplitude.

### Results

**Figure 10.**
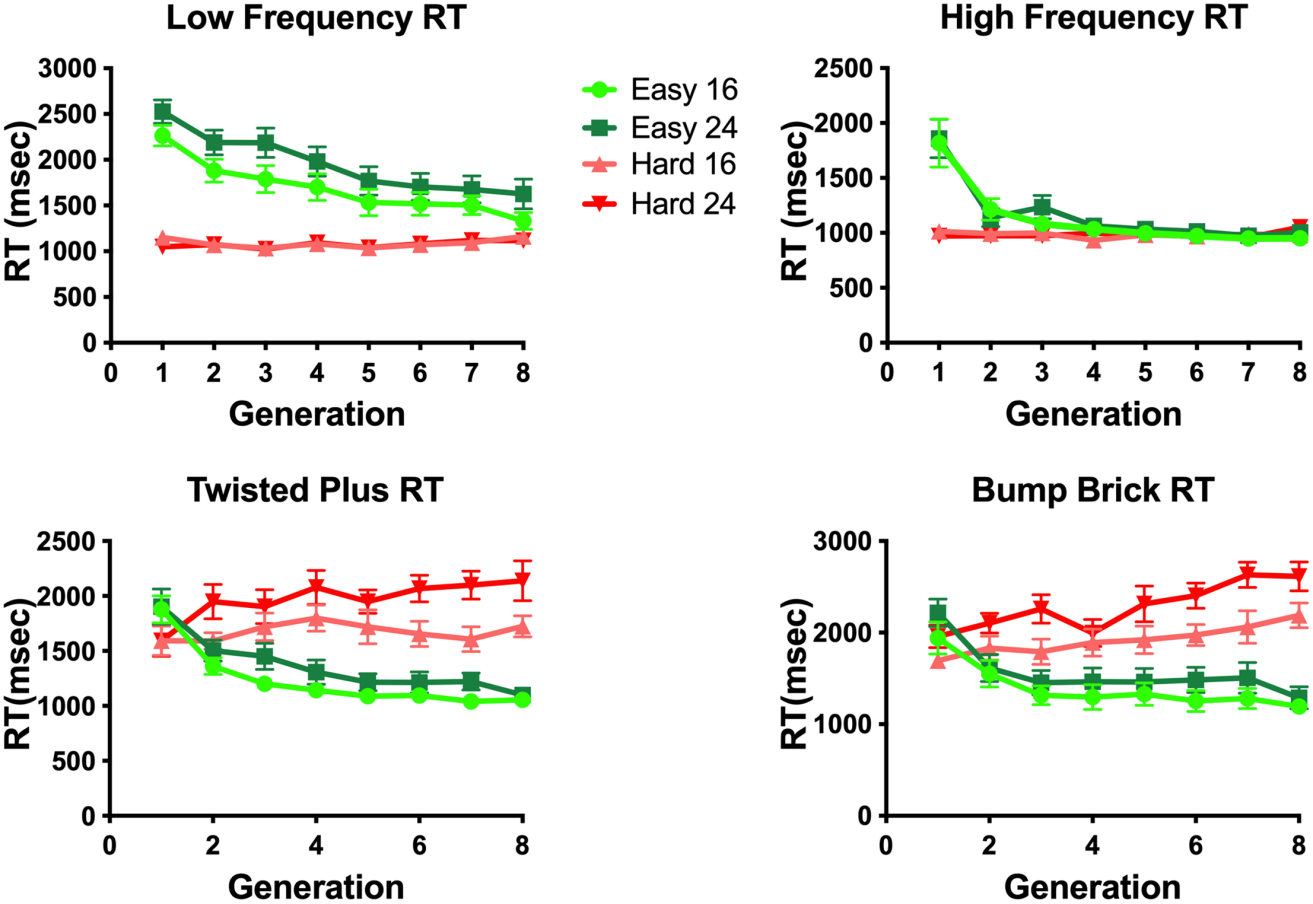
Average RT in msec by generation. Green lines correspond to the evolution direction set to increase the easiness of the search, while red corresponds to increasing the difficulty of the search. Error bars correspond to standard error of the mean.

Figure 10 shows the RTs as a function of generation for each of the four stimulus types. It is clear from the figure that different target shapes produced different results in response to the constraints on the rules for evolution of distractors.

Evolving to make the task easier was possible in all cases. However, searches for the LF and HF targets could not be made significantly more difficult if the distractors were constrained not to contain target frequencies. A 2 (evolution condition) × 4 (target type) repeated-measures ANOVA of the RTs in the first generation shows a main effect of target type (*F*(3,33) = 12.4, *p* < 0.001,$\eta_{G}^{2}=$ .529), a main effect of evolution direction (*F*(1,11) = 79.3, *p* < 0.001,$\eta_{G}^{2}=$ .88), and an interaction between target type and evolution direction (*F*(3,33) = 10.8, *p* < 0.001,$\eta_{G}^{2}=$ .49). As in Experiment 1, these results suggest that there are baseline differences between the search times for the four targets, but in this experiment, there is also an initial baseline difference between the Evolve-Hard and Evolve-Easy conditions. For all four targets, the first generation search times in the Evolve-Easy condition are longer than search times in the Evolve-Hard condition. This can be explained by the frequency constraints: the first-generation distractors are required to match the target on certain frequencies in the Evolve-Easy condition and forced *not* to match the target on certain frequencies in the Evolve-Hard condition. This seems to result in an initial set of “Easy” distractors that are generally harder than the initial “hard” distractors, although the extent to which these groups are different varies across target type.

**Figure 11.**
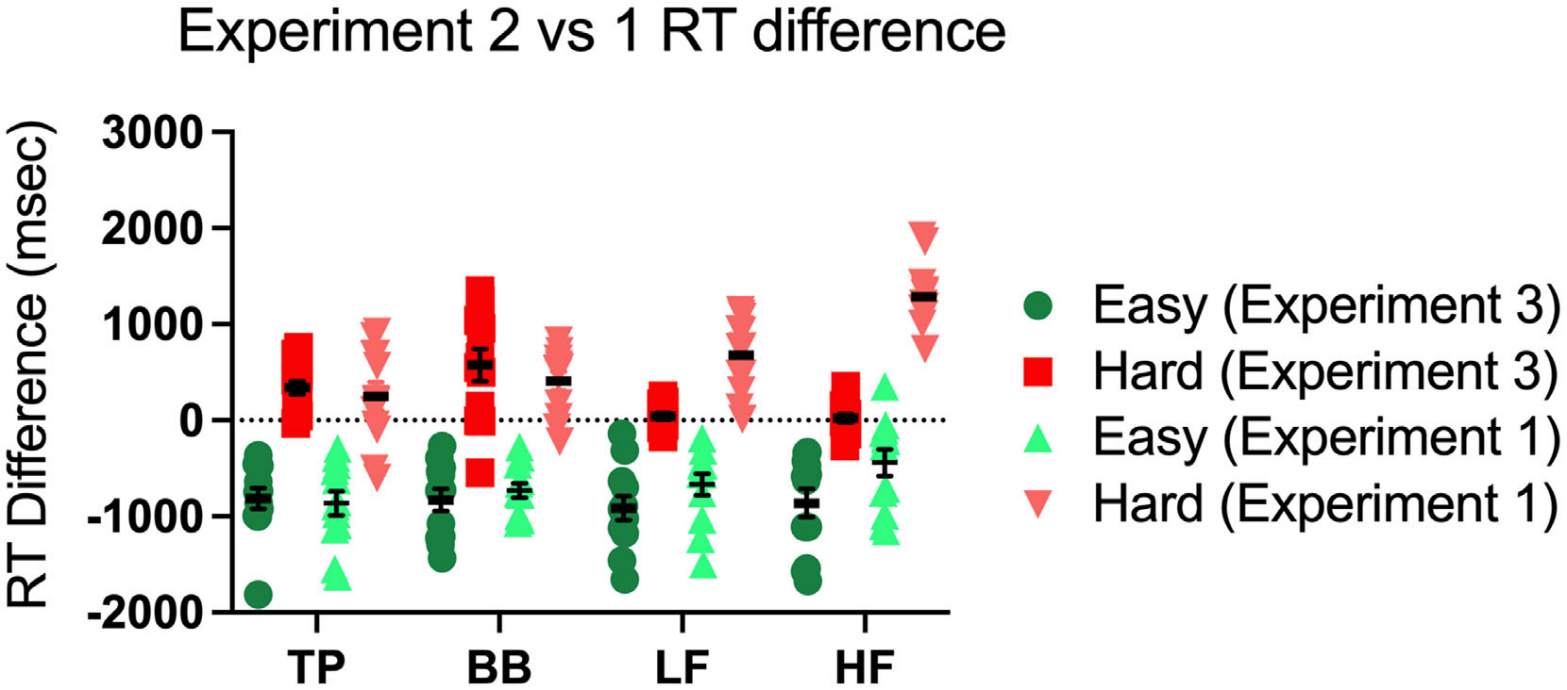
RT difference (generation 8 − generation 1) plotted for all conditions in Experiments 1 and 2. Dark data points correspond to data from Experiment 2, whereas lighter data points correspond to data from Experiment 1. The dotted line represents an RT difference of 0, or no change in RT over eight generations. Data points would be expected to fall on this line if there was no evolution over generations. Error bars correspond to standard error of the mean.

As in Experiment 1, we measured evolution by measuring the difference in RT between generation one to generation eight, shown in Figure 11. A 2 (evolution condition) × 4 (target type) repeated measures ANOVA of the difference in RT shows a significant main effect of evolution direction (*F*(1,11) = 79.3, *p* < 0.001,$\eta_{G}^{2}=0.88$, a significant effect of target type (*F*(3,33) = 12.4, *p* < 0.001,$\eta_{G}^{2}=0.529$), and a significant interaction (*F*(3,33) = 10.8, *p* < 0.001,$\eta_{G}^{2}=0.49$). This suggests that the genetic algorithm is able to produce different distractors in the two evolution conditions, resulting in different changes in RT between the first and last generations.

A one-way repeated measures ANOVA with target type as a factor for the easy condition RT differences reveals no significant difference between target types for the Evolve-Easy condition (*F*(3,33) = 0.22, *p* = 0.90). All the Evolve-Easy conditions produce similar results. The average RT difference for Evolve-Easy blocks, across target types is −855 msec, suggesting that the genetic algorithm is still able to generate distractors that make the search task easier, even when these distractors are forced to contain target frequencies. A one-way repeated measures ANOVA with target type as a factor for the Evolve-Hard condition RTs shows a significant difference between target types for the Evolve-Hard condition (*F*(3,33) = 8.33, *p* < 0.001,$\eta_{G}^{2}=0.43$). As noted, this reflects the inability of the algorithm to make search for the LF and HF targets harder under the constraints of Experiment 2. Post hoc one-sample *t*-tests show that the RT difference in the Evolve-Hard condition is significantly different from zero for the BB and TP targets (*t*(11) = 3.41, *p* < 0.01; *t*(11) = 4.85 *p* < 0.001). However, the RT difference in the Evolve-Hard condition is not significantly different from zero for the HF or LF targets (*t*(11) = 0.49, *p* = 0.63; *t*(11) = 1.13, *p* = 0.28).

**Figure 12.**
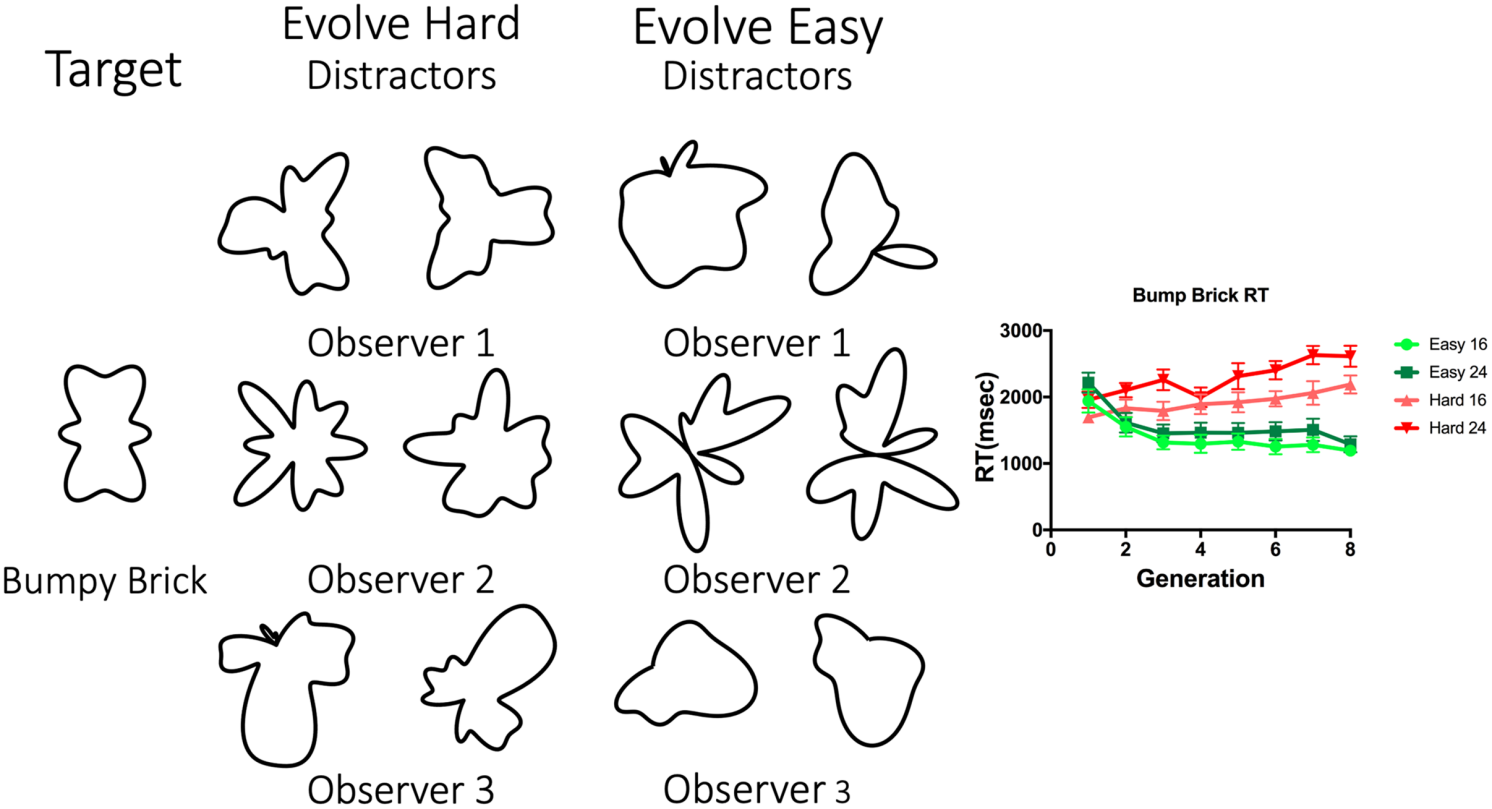
Typical eighth-generation distractors for the Evolve-Easy and Evolve-Hard versions of search for the BB target. RT results are additionally shown; error bars correspond to standard error of the mean.

**Figure 13.**
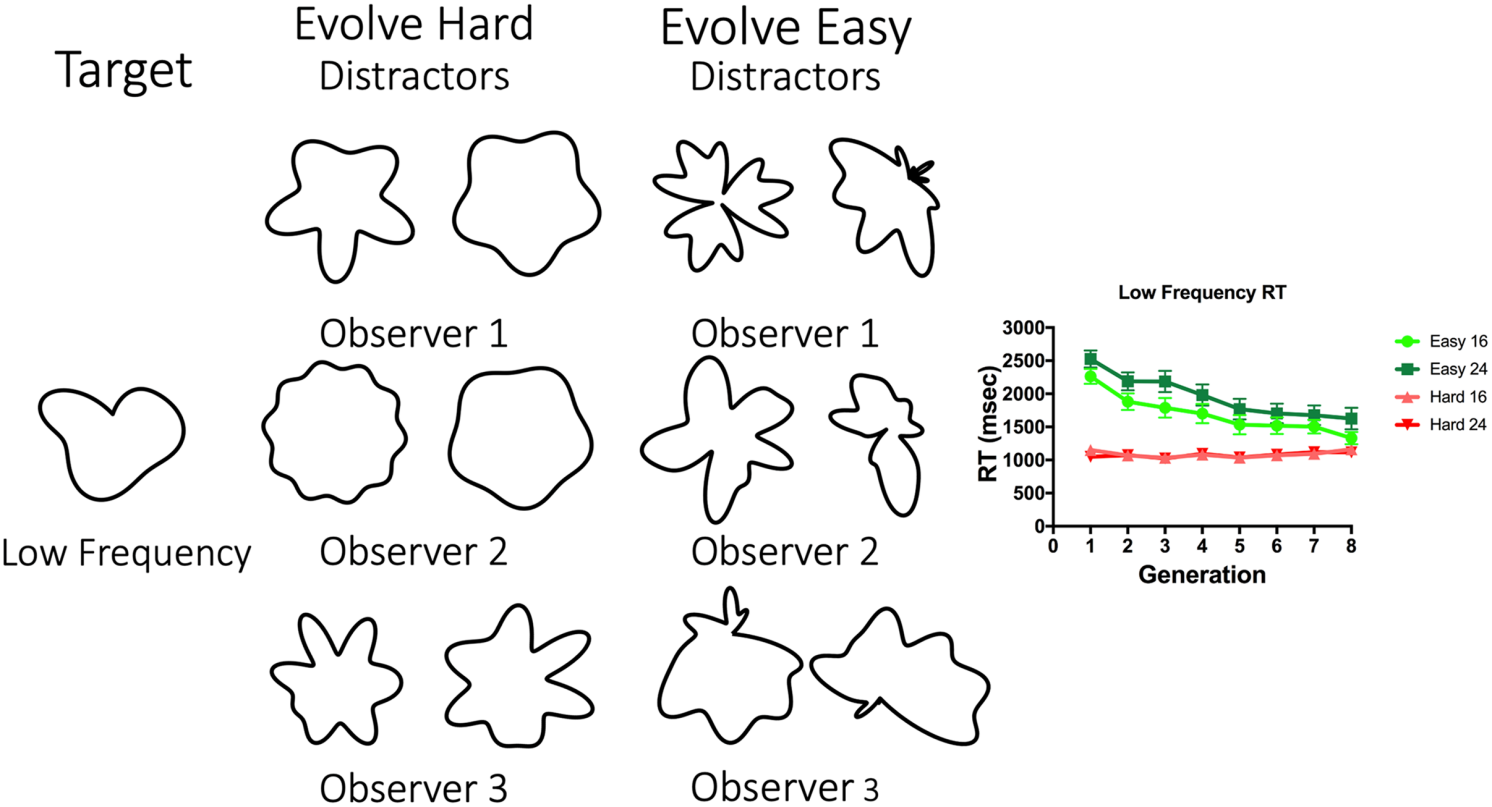
Typical eighth-generation distractors for the Evolve-Easy and Evolve-Hard versions of search for the low frequency target. RT results are additionally shown; error bars correspond to standard error of the mean.

Evolution can make all of these search tasks easier. However, it fails to make the low and high frequency targets harder to find. Figures 12 and 13 give some insight into why this is the case. These figures show sample eighth-generation distractors for three observers each in the Evolve-Easy and Evolve-Hard conditions.

Figure 12 shows results for the BB target. Even though the algorithm is constrained not to use the target frequencies in the Evolve-Hard condition, it creates other bumpy distractors that, although they do not look like the brick, manage to hide it in search. The easy distractors evolve either to a less bumpy blob or, in the case of Observer 2, to a shape that appears to have two parts, again suggesting a role for topology or a parts-based description. Note that each observer's distractors evolve to quite a distinct shape. The algorithm finds different solutions on different runs.

In Figure 13, we see sample results from the LF target. Here the algorithm could make search easy by adding relatively sharp protrusions to the otherwise smooth, low frequency target. Again, the algorithm finds different solutions for different observers. In contrast, constrained not to use the low frequencies that define the low frequency target, the algorithm is unable to make search for that target more difficult. RT is essentially unchanged over the generations because the algorithm fails to eliminate the radial symmetry or sharp points that seem to distinguish the distractors from the target.

Graphs of target-distractor similarity over generations for each target type and evolution condition are shown in Appendix D. The similarity analysis suggests the RT findings are best explained by the radial frequency amplitude distance measurements. The skeleton distance measures showed no change in distractors over generation, and the total curvature distance measure failed to explain key RT findings.

### Discussion

These results suggest that, although observers are searching for a target shape *defined* by a few radial frequencies, the efficiency of their search is not based entirely on the presence or absence of those specific frequencies in the distractors, but on a range of frequencies. It is possible to find distractors that include the target's radial frequencies but nevertheless produce a fast, easy search. And it is possible (although more difficult) to find distractors that exclude the target's radial frequencies but nevertheless produce a slow search. This suggests that the individual radial frequencies present in the search target are not the primary features guiding attention in this shape search task (Wolfe & Horowitz, 2004).

Of course, the presence or absence of the target's component frequencies in the distractors will have some effect on the difficulty of the search. This can be seen in the first block of the search task: randomly-generated distractors that contain the target's radial frequencies generally produce slower search times than randomly-generated distractors that exclude the target's radial frequencies. As in Experiment 1, search times seem to be related to the overall similarity between the target and distractor shapes’ amplitude spectra: the initial “easy” distractors are more similar to the target (and thus produce slower search) whereas the initial “hard” distractors are dissimilar from the target (and thus produce a faster search).

Similarity in amplitude spectra may also explain why it was more difficult to evolve “hard” than “easy” distractors. When the distractors cannot include the target frequencies, some paths to target-distractor similarity are blocked. Nevertheless, as shown in Figure 12, properties of shape like “bumpiness” can be generated in various different ways to make distractors that behave as though they are similar to the target even without the target frequencies. Going in the other direction, even when the distractors are forced to include the target frequencies, it is still possible to produce dissimilar distractors by evolving high amplitude on other frequencies. These findings were best captured by the radial frequency target-distractor difference metric rather than a skeleton or curvature metric.

## Experiment 3: Hiding the rabbit

The previous experiments used meaningless abstract shapes as targets and distractors. In Experiment 3 we investigate whether the same genetic algorithm approach can be used to generate hard or easy distractors for a semantically meaningful natural shape target. Natural shapes have very different statistics from radial basis patterns (Schmidtmann & Fruend, 2019), and representing a natural shape as a radial basis pattern generally requires more than 10 radial frequencies. However, Experiment 2 suggests that it is possible to hide a target among distractors that do not include those spatial frequencies, so it might be possible to hide a natural shape among simpler radial basis pattern distractors.

### Methods

Data were collected from 12 new participants (eight females, four males, mean age 27.4 years). All observers had normal or corrected-to-normal vision and passed the Ishihara test for color vision. All experimental procedures were approved by the Brigham and Women's Hospital Institutional Review Board, and all participants gave informed consent and were paid $10 an hour.

### Apparatus and stimuli

**Figure 14.**
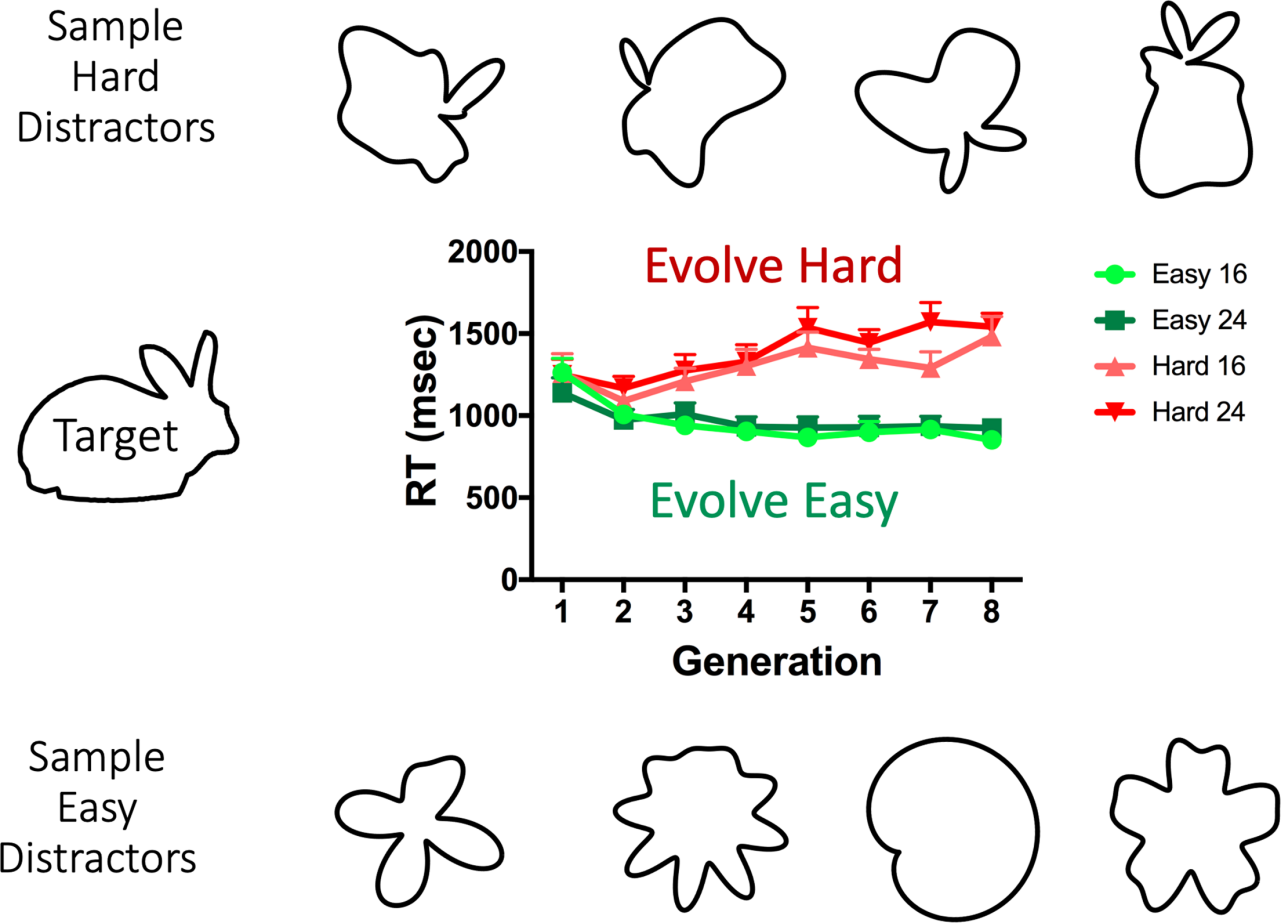
Rabbit target and sample easy and hard distractors. Graph shows RT as a function of generation for the Evolve-Easy and Evolve-Hard conditions in two set sizes. Error bars correspond to standard error of the mean.

The search task was similar to previous experiments, but there was only one target, a rabbit silhouette. This shape was chosen because it was a recognizable object which could be represented as a radial basis pattern. The rabbit shape is defined by 360 points along its circumference, so an exact representation of this target requires 359 radial frequencies. An example of the rabbit shape target is shown in Figure 14. As in previous experiments, participants searched for this target over eight blocks in each of two conditions (Evolve-Easy and Evolve-Hard) with the order of conditions counterbalanced across observers.

### Results

Figure 14 shows the average RT over generations split by evolution direction and set size. A 2 (evolution direction) × 8 (generation) repeated measures ANOVA shows a main effect of evolution direction (*F*(1,11) = 30.18, *p* < 0.001, $\eta_{G}^{2}=0.73$). There is no main effect of generation (*F*(1,11) = 0.21, *p* < 0.65) but there is a significant interaction between evolution direction and generation (*F*(1,11) = 18.21, *p* = 0.001,$\eta_{G}^{2}=0.62$) because Evolve-Hard becomes slower whereas Evolve-Easy becomes faster. A post-hoc *t*-test over the first generation RTs shows there is no significant difference between Evolve-Easy and Evolve-Hard RTs in the first generation (*t*(11) = 0.48, *p* = 0.64). This is expected, because the initial distractors in both evolution conditions are random. However, a *t*-test over RTs in generation 8 shows a significant difference between Evolve-Easy and Evolve-Hard RTs (*t*(11) = 9.67, *p* < 0.0001). As with semantically meaningless stimuli, the genetic algorithm approach is able to evolve easy and hard distractors for a semantically meaningful natural shape target.

The sample distractors in Figure 14 give some intuition about what is evolving in this experiment. The bunny is an elongated blob with ears. The hard distractors seem to capture those aspects of the shape while looking nothing like a rabbit. The easy distractors tend to be too radially symmetric or too smooth to interfere with search for the rabbit target.

In this case, the effects of evolution seem to be best captured by the shape skeleton measure from Ayzenberg and Lourenco (2019). This may be intuitively clear from inspection of Figure 14, where it seems that the hard distractors have a main axis and a part or two whereas the easy distractors lack that main axis. The effects of evolution were not obvious in differences in target and distractor amplitude spectra or total curvature (Details of this analysis are shown in appendix E).

### Discussion

Although the genetic algorithm is able to create hard distractors for the rabbit silhouette target, search times in the final generation of the Evolve-Hard condition remain faster (average 1512 msec) than the search times in the final Evolve-Hard generation of any of the abstract shape targets (the fastest of these, target TP, had a final average RT of 1971 msec in the Evolve-Hard condition). This may be partly due to the fact that the distractors did not contain the higher radial frequencies present in the rabbit shape. So, whatever result the algorithm creates over the first 10 frequencies, the higher frequencies always remain uniquely present in the rabbit and may aid search. It is all the more striking, therefore, how the genetic algorithm finds shapes that make it harder to find the rabbit.

Rabbit search efficiency is not based on semantic meaning of the rabbit target. The distractor shapes that effectively hide the rabbit don't particularly resemble rabbits, and it's unlikely that any of them would be labeled “rabbits” by a naïve observer. The part structure of the distractors appears to be relevant in the search for the rabbit, whereas the semantic meaning (“Rabbit”) is not. The distractors that most effectively hide the rabbit shape target seem to have roughly replicated the target components of a larger “body” and one or more narrow ear-like tufts. It is unclear whether a single part of the item (e.g., a rabbit-like body *or* rabbit-like ears, alone) is sufficient to effectively hide the rabbit target. Alternatively, the part-whole relationship of these items (a blob with ears) could be the critical factor that makes these distractors effective. One way to address this issue is to create distractors with extra “ears.” If the ears are the critical feature, multi-eared objects might be particularly effective distractors. If the holistic configuration of rabbit-like body plus rabbit-like ears is important, then ear-enriched distractors might be more easily disregarded, and search might be easier. We test this hypothesis in a final experiment.

## Experiment 4

### Methods

Data were collected from 12 participants (nine females, three males, mean age 20 years). All observers had normal or corrected-to-normal vision and passed the Ishihara test for color vision. All experimental procedures were approved by the Brigham and Women's Hospital Institutional Review Board, and all participants gave informed consent and were paid $10 an hour.

### Apparatus and stimuli

**Figure 15.**
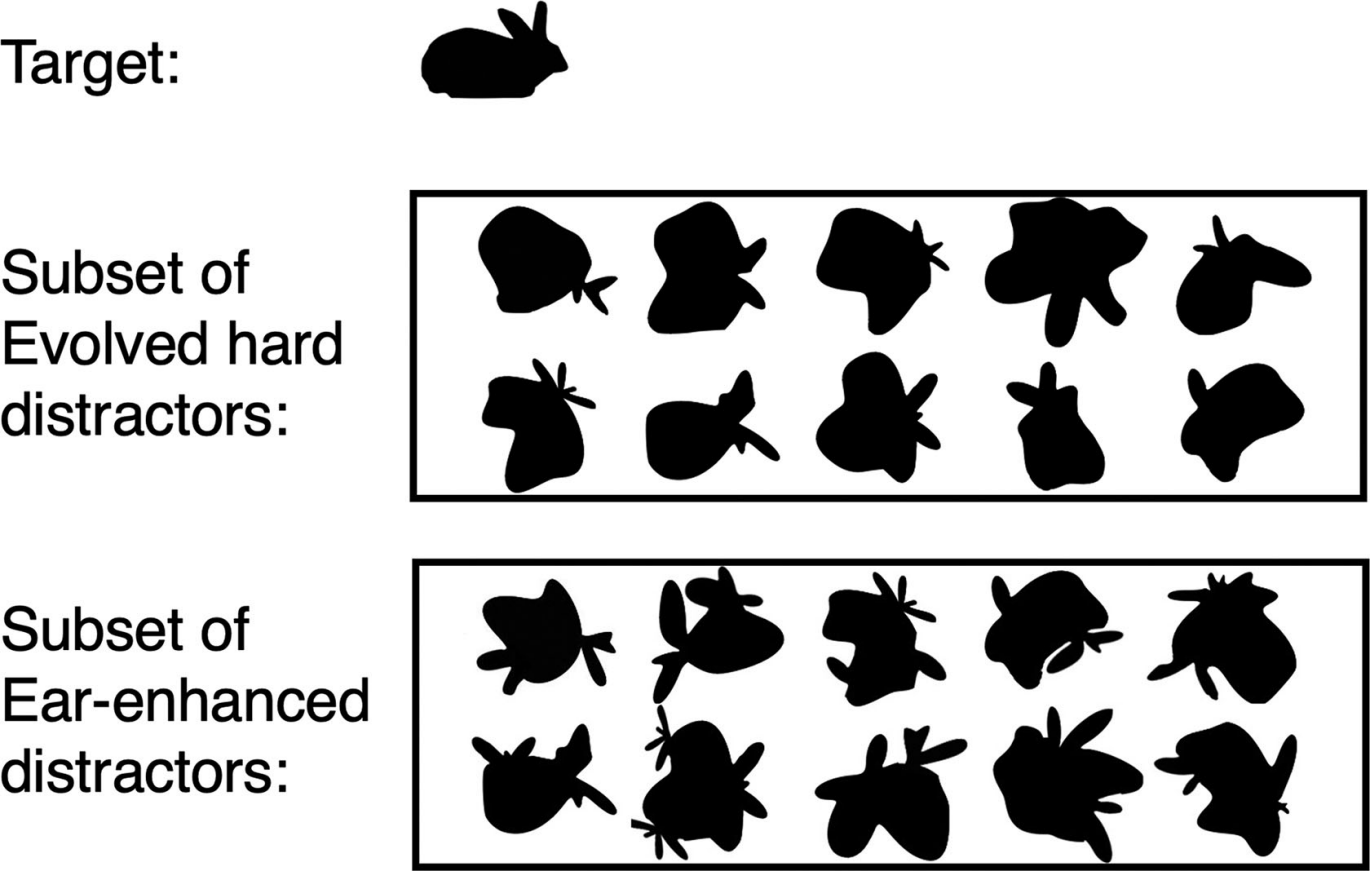
Stimuli for both search conditions in Experiment 4.

To create distractors with extra parts, we randomly selected 10 of the Evolve-Hard distractors that were effective in slowing search for the rabbit target. We manually added extra “ears” to each of these distractors by copying the narrow ear-like tuft parts from other distractors and pasting these onto the “body” of the distractor. Additionally, 10 different Evolve-Hard distractors were chosen to use unaltered as distractors. The Evolve-Hard and the ear-enhanced distractors are shown in Figure 15.

The size and orientation of all items in the search displays were randomly jittered to prevent the rabbit from being detected as an item of specific size or specific orientation. Orientations were constrained to ±25° to ensure that the rabbit always appeared to be upright, even when tilted.

### Procedure

The two distractor conditions, Evolve-Hard and Ear-Enhanced, were shown in separate blocks, with block order counterbalanced across observers. Each block consisted of 20 practice trials, followed by 300 experimental trials. On each trial, observers searched for the rabbit silhouette target among a homogeneous array of distractors with set size 8, 12, or 16 items. The rabbit target was present in 50% of trials. Observers indicated whether the target was present or absent by a keypress.

### Results

**Figure 16.**
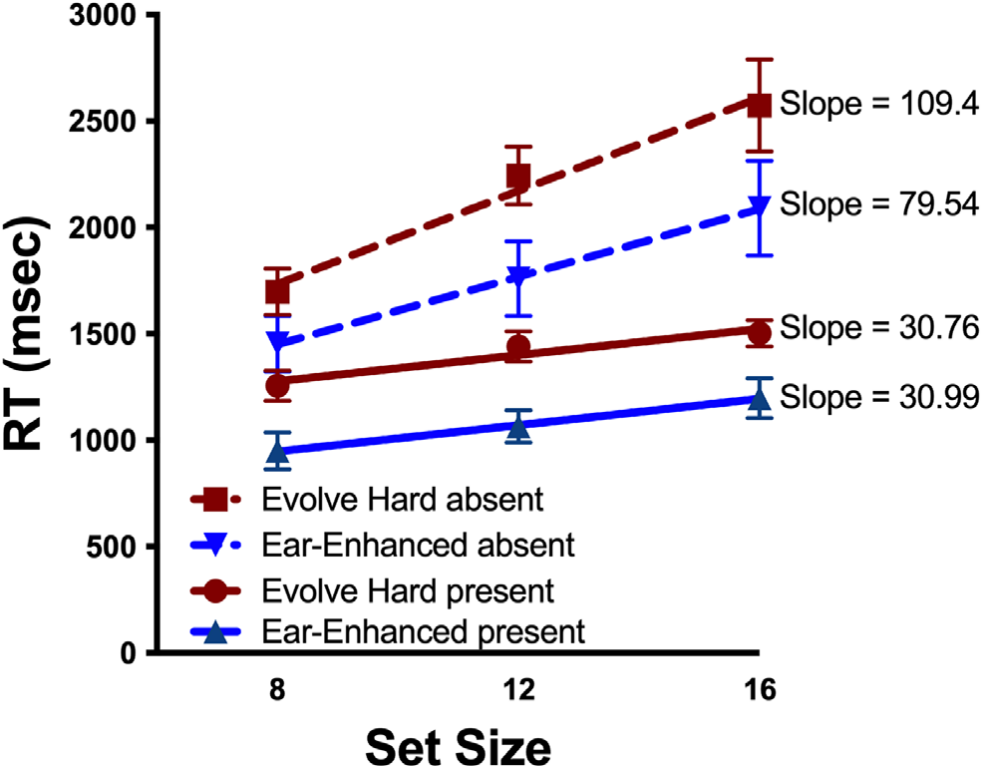
RT by set size functions for each condition. Dotted lines correspond to target absent trials while solid lines correspond to target present trials. Lines plotted show a linear fit, and error bars correspond to standard error of the mean.

Average search slopes were computed from the mean RTs of each observer. RTs greater than 10,000 msec were excluded from analysis as were RTs from the practice trials. Figure 16 shows average RT by set size for target-present and target-absent trials in each distractor condition. As can be seen in the figure, the Ear-enhanced condition produces faster, but not more efficient search. A comparison of the search slopes reveals that the Evolve-Hard and Ear-Enhanced distractors produced similarly inefficient search.

Figure 16 shows that adding extra “ears” reduced RTs. This effect appears to be additive, without an effect on the slope of RT x set size functions. A 2 (distractor condition) × 3 (set size) repeated measures ANOVA over RT for target present trials shows a main effect of distractor condition (*F*(1,11) = 11.96, *p* = 0.005, $\eta_{G}^{2}=0.521$), a main effect of set size (*F*(2,22) = 40.10, *p* < 0.001, $\eta_{G}^{2}=0.79$), with no interaction (*F*(2,22) = .85, *p* = 0.44, $\eta_{G}^{2}=0.07$). A 2 (distractor condition) × 3 (set size) × repeated measures ANOVA over RT for target absent trials falls short of showing a main effect of distractor condition (*F*(1,11) = 4.09, *p* = 0.07, $\eta_{G}^{2}=0.521$), shows a main effect of set size (*F*(2,22) = 41.73, *p* < 0.001, $\eta_{G}^{2}=0.79$), with an interaction falling short of statistical significance (*F*(2,22) = 0.325, *p* = 0.058, $\eta_{G}^{2}=0.23$). Paired *t*-tests comparing target present RT × set size slopes for evolved hard distractors (mean slope = 30.76) and ear-enhanced distractors (mean slope = 30.99) did not find a significant difference between the two (*t*(11) = .06, *p* = .95). Paired *t*-tests comparing target absent RT × set size slopes for evolved hard distractors (mean slope = 109.4) and ear-enhanced distractors (mean slope = 79.54) did not reach significance (*t*(11) = 1.83, *p* = 0.05). As ever, target absent conditions facilitate a longer search duration than target present conditions.

### Discussion

The effect of adding extra “ears,” or narrow elongated parts, to the blob with ears distractors reduced RTs while having little effect on the slope of RT × set size functions. Although there are many ways to interpret visual search data of this sort, one standard interpretation of this pattern of results would be to argue that the addition of extra ears did not affect the search process but speeded the final decision stage. That is, both sets of distractors produced a relatively inefficient search, consistent with serial selection of one candidate rabbit after the other. On target present trials, when the rabbit is eventually selected, the decision that the selected item is the target is faster when the distractors have extra ears. Absent trials are sensitive to the time required to make a present response (Chun & Wolfe, 1996; Moran, Zehetleitner, Müller, & Usher, 2013), and, as a consequence, the absent RTs are also somewhat shorter for multi-ear distractors.

If extra ears had made it easier to reject distractors, then the slopes of RT × set size functions would be expected to be shallower. Similarly, if observers were making faster decisions about each distractor during search, one would expect to see a shallower slope for the multi-ear distractors. The lack of a slope effect suggests that attention is not being guided by the number of ears. Targets with one or two ears do not pop-out among distractors with four or more. It is possible that there would be a search asymmetry here. Targets with more of an attribute are often easier to find than targets with less (Treisman, 1985; Wolfe, 2001). Thus it could be that multi-eared distractors would pop-out from amongst rabbit or rabbit-like distractors.

Overall, these results are consistent with an account in which guidance to the rabbit silhouette is difficult among distractors that are composed of a body plus ears, even supernumerary ears. The speed with which one can make a final response about the presence or absence of a rabbit depends on the similarity of the target and the attended distractors, but this effect does not influence the aspects of the search process that depend on the number of items in the search display.

## General discussion

What have we learned from these experiments?

### The method works as intended

These experiments show that a GA can be used to increase or decrease the difficulty of a visual search based on an observers’ own behavior, even with a relatively noisy metric of evolutionary fitness such as RT. These studies suggest that GAs can be used to make search more or less efficient. These experiments can be thought of as a proof of concept, showing that the GA method has promise. We have by no means exhausted the possibilities here. It is certainly possible that another set of “genes” would produce different, novel results. Radial frequency descriptors are convenient because they are easy to parameterize, but they generate only a fairly restricted set of shapes (fairly schematic rabbits—yes; trees, Swiss cheese, or Chinese characters—not so readily). Other descriptors like, for example, medial-axis skeletons (Ayzenberg & Lourenco, 2019) or parameters derived from an object-recognizing neural net (Kreiman, 2017) might produce interesting and quite different results. Hung, Carlson & Connor (2012) used an adaptive shape sampling approach to demonstrate that macaque monkey IT encodes medial axis shape, which suggests that focusing on medial-axis skeletons may be a promising future direction for more complex shapes.

Even within the radial frequency space, different evolutionary rules might produce different results. For instance, asexual reproduction might produce different trajectories of evolution. A richer family of shapes might yield different guiding shape features. Hiding a rabbit did not require higher radial frequencies, but would that be true if we tried to hide a simple square or an “X”? Our algorithm was initialized by randomly selecting 10 amplitudes and dividing each by the respective frequency to approximate the spectrum of natural scenes. It's unclear whether the decision to approximate the 1/f rule for the Fourier spectra of real scenes (Black, Hurst, & Simaika, 1965) influenced the pattern on shape evolution. In short, the basic success of the GA method suggests that a range of follow-on experiments could produce useful results.

### Radial frequencies are a useful but not a comprehensive description of shape

As noted above, radial frequencies can be used to generate a range, but by no means the *full* range of shapes and the GA method can manipulate radial frequencies to make easier and harder searches for specific targets. Experiment 2 forced distractors to include or exclude the radial frequencies of the target. The results of evolution found that it was possible to find difficult distractors for some target shapes but not others. When the target was defined by a few discrete frequencies, search could be made difficult by evolving distractors to include other, neighboring frequencies. Search could be made easy by evolving distractors that were quite simple blobs among targets with more apparent “parts.” However, when the target was defined by a range of high or low frequencies, it was not possible to make the search harder when distractors could not include the same frequencies as the target. High-radial-frequency distractors simply could not hide low-radial-frequency targets or vice versa. This result is reminiscent of classic spatial frequency channel psychophysics in which high frequency targets were not masked by low frequency noise and vice versa (Legge & Foley, 1980).

### A radial frequency-based GA suggests a role for part-whole accounts of shape

Experiments 3 and 4 revealed the GA's ability to hide a semantically meaningful shape like a rabbit-silhouette target, even though the rabbit includes high frequency components not present in the distractors. Interestingly, the distractors that most effectively hide the rabbit do not have the appearance of a rabbit. Moreover, a shared radial frequency account does not seem to do very much work in explaining what makes some rabbit searches harder than others. Instead, the GA appears to have “discovered” evidence for accounts of shape that focus on the part structure of shapes. The primary apparent similarity among the best rabbit-hiding distractors is that they all seem to be comprised of a combination of a larger, elongated body-like part and one or more narrow ear-like parts. The exact contours of the “body” and placement of the “ears” on that body do not seem to be critical. Again, there is no requirement that the distractor look like a rabbit. Adding extra “ears” to the distractor shape (Experiment 4) does not seem to influence the search process but does seem to make it easier for observers to decide when they have found a target rabbit.

In order to parameterize the target and distractor shapes, and to describe target-distractor similarity with evolution, three metrics were used - a shape skeleton similarity metric, a measure of total curvature (perimeter^2^/area), and a Fourier amplitude distance measure based on the radial frequency spectrum. Given that the experiments manipulated radial frequency, it is, perhaps, not surprising that the Fourier amplitude measure succeeded in capturing the evolution from easy to hard or vice versa in, for example, Experiment 1. It is more interesting that it failed to explain the changes that produced distractor shapes that hid the rabbit target (Experiments 3, 4). The skeleton similarity metric better described these distractor changes, presumably because skeletons capture the part-whole structures that evolved when we tried to hide the rabbit.

### The results are consistent with the existence of two types of “Template” in search

*Finally,* these findings illustrate something important about the role of templates in visual search. A search target like a rabbit or a “twisted plus” must be represented in the mind of the observer. These representations are often called “templates.” There is considerable discussion of templates in current search literature. Several terms are used, essentially interchangeably. Template names include the search template (Rajsic, Ouslis, Wilson, & Pratt, 2017), the memory template (T. Kristjánsson, Thornton, & Kristjánsson, 2018), the target template (Bravo & Farid, 2016), and the attentional template (Yu & Geng, 2019). The current results serve as an illustration that these terms actually refer to two different types of templates (Wolfe, 2020; Wolfe, 2021). Return to the “twisted plus” for an example. How do we guide attention to a “twisted plus” target? Apparently, for search purposes, it is defined as something like a “roughly round (not elongated) thing with bumps.” Search for this target is difficult when distractors are roughly round and bumpy, while search is easy when distractors are elongated, smooth, or both. We could speak of a “guiding template” that embodies this definition of the target of search. Once an item is selected, however, this guiding template is not adequate to identify the target as, specifically, the twisted plus. There must be a second “target template” that contains the precise representation of the target. This is the representation that allows you to distinguish your child from other children or your small red car from other small red cars.

This point may be made most clearly by Experiment 4. In both the regular and extra ear conditions, the distractors appear to match the rough *guiding* template of elongated body plus ears. Search is inefficient because target and distractors share the features of that guiding template. Neither type of distractor matches the *target* template that identifies This Specific Rabbit. The mismatch is greater for the extra ear distractors, allowing positive identification of the target to occur more quickly in the extra ear condition. The two-template idea is a different way to account for the ability to hide a target with distractors that, once attended to, look nothing like the target. The target is hidden in search if distractors match the guiding template. It is identified when selected, if the target matches the target template.

If a target has a specific orientation, the guiding template will have a rough, one-dimensional representation of that orientation. It would have a three-dimensional representation of the target's color. The guiding representation of the target's shape may be of quite a high dimensionality. The results of the present experiments point to frequency dimensions, part-whole/ skeletal dimensions, as well as the topological dimensions that made it easy to find a target with an internal loop. It may be that there is no single description that combines all of these into a unified description of shape. This venture into using a GA to study shape in search did not reveal any such new representation. These experiments did demonstrate that GA's can be used in this quest. Other implementations of GAs have been used to optimize stimuli (Verma & McOwan, 2009), optimally simulate human search behavior (Zhang & Eckstein, 2010), and to explore which features constrain search performance in a search display (Van der Burg, Cass, Theeuwes, & Alais, 2015). Future work using GAs may extract more information about the shape feature space that undergirds guidance of attention by shape information.

## Supplementary Material

Supplement 1
