# Supplementary material for: Hiding the Rabbit: Using a genetic algorithm to investigate shape guidance in visual search
Source: J Vis. 2022 Jan 13;22(1):7. doi: 10.1167/jov.22.1.7 (PMC8762685; doi:10.1167/jov.22.1.7)
Supplement: Supplement 1 [file jovi-22-1-7_s001.pdf]

Supplemental Materials

Appendix A:

Each distractor shape d is defined by a vector of phases $\phi_d = \langle \phi_{d1}, \phi_{d2}, \dots, \phi_{d10} \rangle$, a vector of amplitudes $a_d = \langle a_{d1}, a_{d2}, \dots, a_{d10} \rangle$, and a binary vector $b_d = \langle b_{d1}, b_{d2}, \dots, b_{d10} \rangle$, which determines whether a given radial frequency is on or off (off means the amplitude is replaced by 0).

To find the parameters for an "offspring" shape, two "parents" are randomly selected from the top three distractors of the previous generation. Denote the parent shapes as x and y with parameters $\{\phi_x, a_x, b_x\}$ and $\{\phi_y, a_y, b_y\}$. Offspring parameters $\{\phi_w, a_w, b_w\}$ are a function of the parent parameters and mutation rate m $(0 < m < 1)$ as follows:

$$\phi_{\omega i} = \begin{cases} \phi_{xi} & \text{with prob. } p = (1\text{-}m)/2 \\ \phi_{yi} & \text{with prob. } p = (1\text{-}m)/2 \\ \text{drawn uniformly from the range } (0, 2\pi] & \text{with prob. } p = m \end{cases} \quad 1)$$

$$a_{\omega i} = \begin{cases} a_{xi} & \text{with prob. } p = (1\text{-}m)/2 \\ a_{yi} & \text{with prob. } p = (1\text{-}m)/2 \\ \text{drawn uniformly from the set } \{2a_{xi}, 2a_{yi}, \frac{a_{xi}}{2}, \frac{a_{yi}}{2}\} & \text{with prob. } p = m \end{cases} \quad 2)$$

$$b_{\omega i} = \begin{cases} b_{xi} & \text{with prob. } p = (1\text{-}m)/2 \\ b_{yi} & \text{with prob. } p = (1\text{-}m)/2 \\ \text{drawn uniformly from the set } \{(1\text{-}b_{xi}), (1\text{-}b_{yi})\} & \text{with prob. } p = m \end{cases} \quad 3)$$

The selection of "parents" is repeated (with replacement) to generate the required number of "offspring" shapes.

Appendix B:

We quantified the similarity between target and distractor shapes by using a few different shape parametrization methods

Skeleton similarity:

We used the *bwmorph* function in MATLAB, which generates skeletons derived from morpholoical thinning. The skeleton similarity metric from Ayzenberg & Lourenco (2019) was used to calculate the difference between target and distractor images. The results from this analysis are shown in Figure B1. A 2 (evolution condition) x 4 (target type) ANOVA shows a significant main effect of evolution direction ($F(1,11) = 63.02$, $p < 0.01$), a significant main effect of target ($F(3,33) = 4.710$, $p < 0.01$), and no interaction ($F(3,33) = 1.111$, $p = 0.36$).

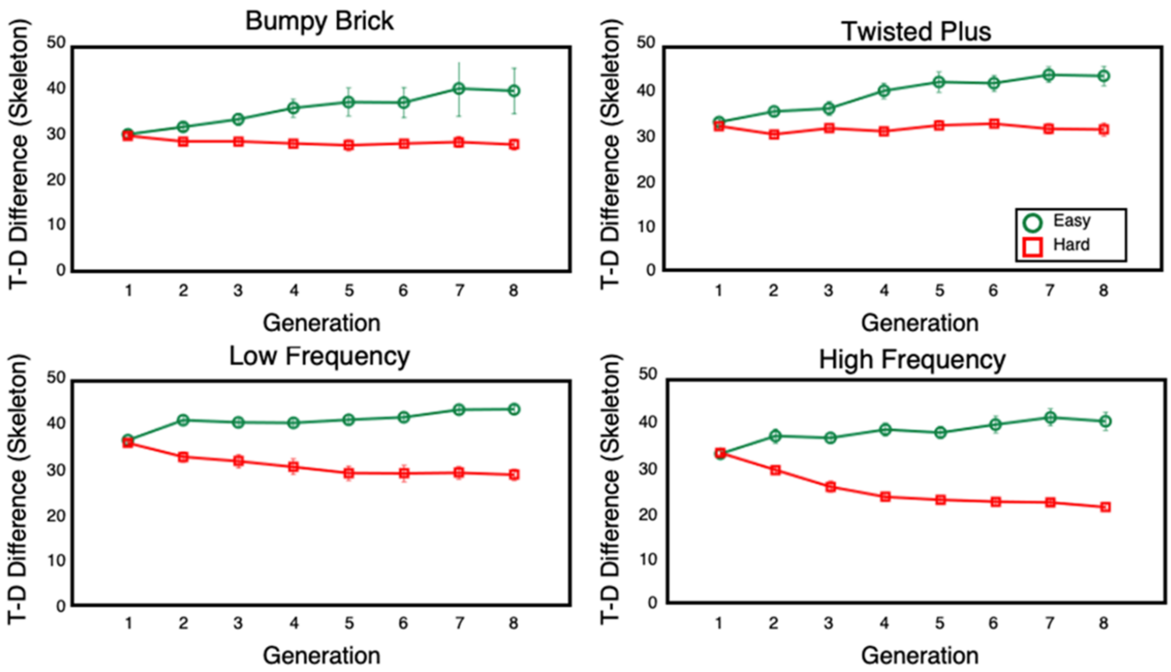

Figure B1. Target-distractor difference between shape skeletons across generations for each target type and evolution condition. Error bars correspond to S.E.M.

Total curvature (perimeter^2/area):

We calculated the perimeter and area of the target and distractor shapes, and for each shape computed the perimeter^2/area. The average target-distractor difference over generations for each

of the four target types is shown in figure B2. A 2 (evolution condition) x 4 (target type) repeated measures ANOVA of the difference between the similarity in the first and last generation does not quite reach significance for the main effect of evolution condition ($F(1,11) = 3.66$, p =0.08,), a main effect of target type ($F(3,33) = 3.96$, p =0.015), and a significant interaction ($F(3,33)$ = 18.01, $p < 0.001$). Bonferroni-corrected post-hoc tests, performed between the Evolve-Hard and Evolve-Easy conditions, show significant pairwise differences for all targets (all $p < .009$) except the BB target ($p = .19$). These results suggest a larger perimeter^2/area is needed to hide a target, except in the Low Frequency case. A spiky distractor, or one with many high frequency parts will have a greater perimeter^2/area, and amongst such a distractor it would be easy to find a low frequency target.

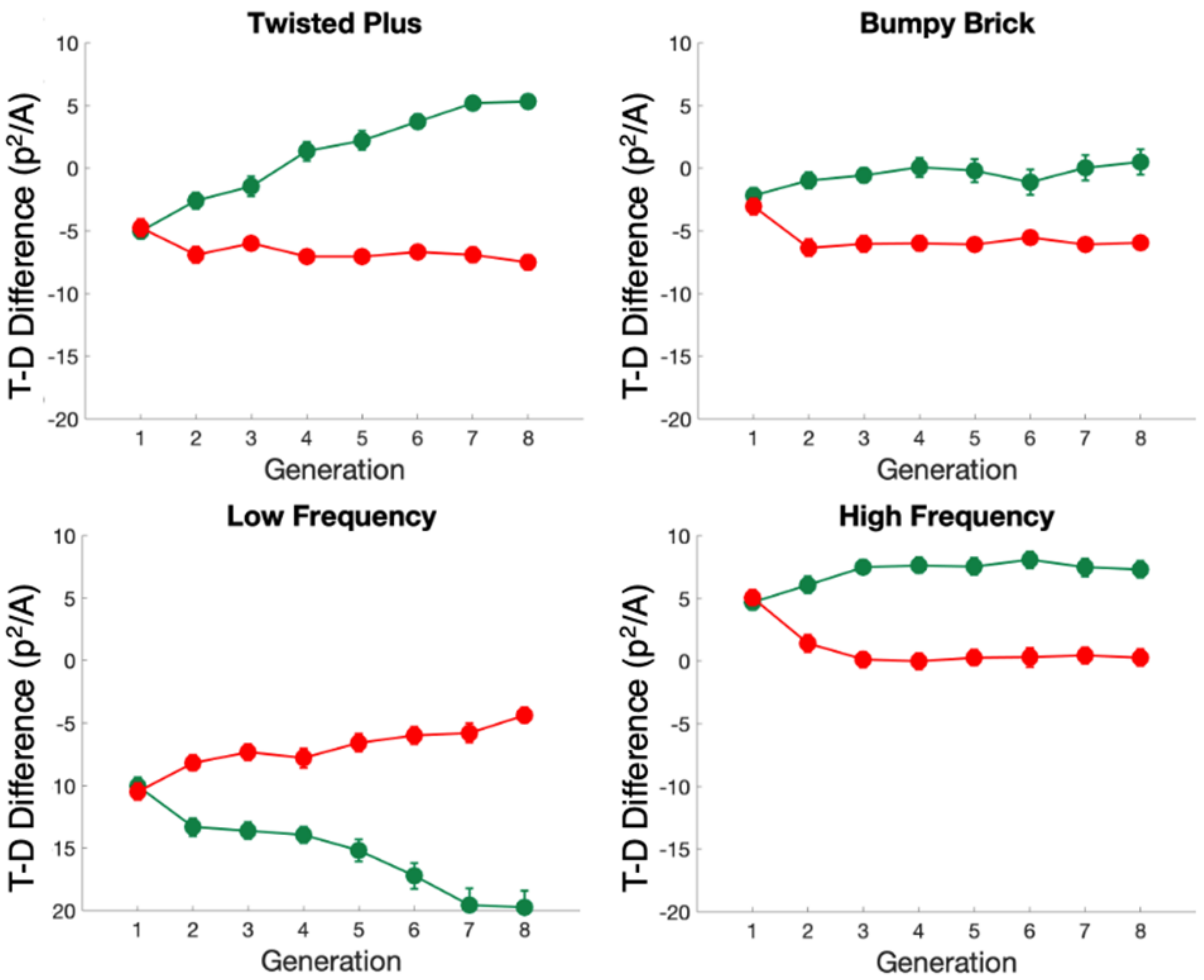

Figure B2. Target-distractor difference of total curvature (measured as perimeter^2/area) . Error bars correspond to S.E.M.

Fourier amplitude spectra:

We quantified the similarity between target and distractor shapes by computing the Euclidian distance (L2 norm) between their Fourier amplitudes across the ten frequencies, which were normalized to sum to 1. The average target-distractor similarity over generation for the four target types is shown in Figure B3. In the Evolve-Hard conditions, distractors tend to become more similar to the target (the difference in their Fourier amplitudes decreased) and in the Evolve-Easy conditions, distractors tend to become less similar to the target. A 2 (evolution condition) x 4 (target type) repeated measures ANOVA of the difference between the similarity in the first and last generation shows a main effect of evolution condition ($F(1,11) = 113.17$, $p < 0.001, \eta_G^2 = .61$), a main effect of target type ($F(3,33) = 5.26$, $p < 0.01, \eta_G^2 = .16$), and a significant interaction ($F(3,33) = 6.47$, $p < 0.01, \eta_G^2 = .15$). This suggests that the two evolution conditions (Evolve-Easy and Evolve-Hard) produce distractors that are different in terms of their similarity to the target. The size of this difference varies across the four target types. Bonferroni-corrected post-hoc tests, performed separately within the Evolve-Hard and Evolve-Easy conditions, show significant pairwise differences only in the Evolve-Easy condition, between the BB and TP targets (($t(11) = 4.10$, $p < 0.05$) and between the BB and LF targets ($t(11) = 6.15$, $p < 0.001$).

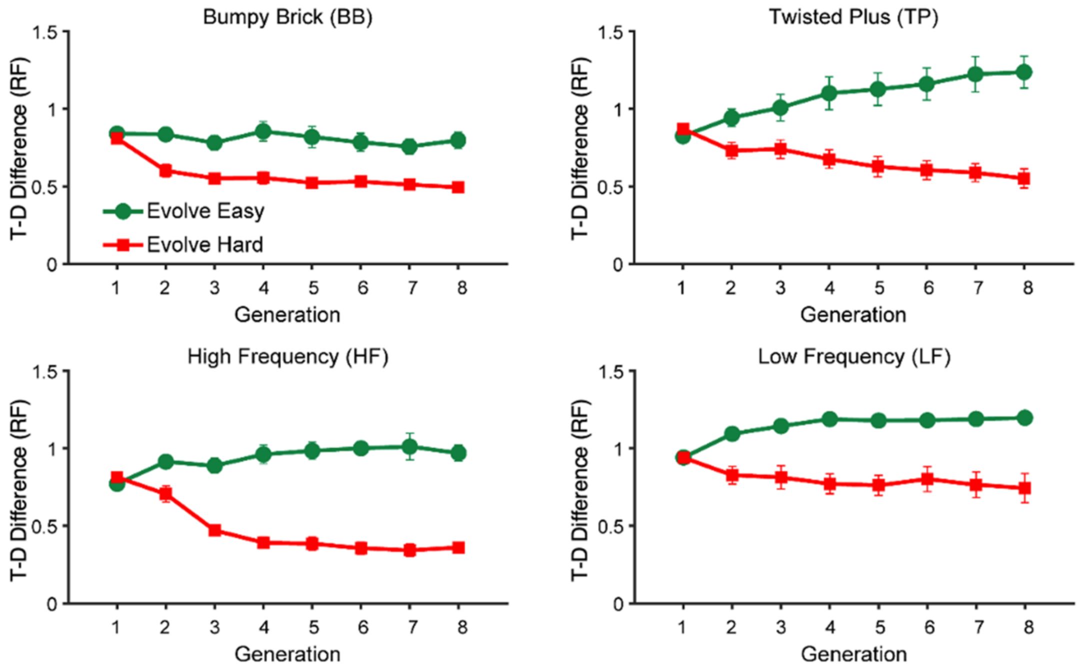

Figure B3. Average target-distractor difference (measured as the L2 norm distance between their Fourier amplitude spectra) across generations for each target type and evolution condition. Error bars correspond to S.E.M.

Another question that arises is whether the GA converges upon a single solution. Does every evolution sequence settle on similar "hardest" and "easiest" distractor "genomes?" To quantify the degree to which distractors became more similar to each other, we took all the distractors from a single generation and computed the L2 norm distances between their individual amplitude spectra and the average amplitude spectrum of the group. We measured this separately for each target and each evolution direction. The results are shown in Figure B4. For most targets, the Evolve-Hard distractors become more homogenous than the Evolve-Easy distractors over generations while the Evolve-Easy distractors stay fairly inhomogeneous (similar to the initial, random distractor population), however the LF target shows the opposite result. A 2 (evolution condition) x 4 (target type) repeated measures ANOVA of the difference from the mean amplitude spectra shows a main effect of evolution condition ($F(1,11) = 11.44$, $p < .01, \eta^2_G = .16$), no significant effect of target type ($F(3,33) = 0.42$, $p = .74$), and a significant interaction ($F(3,33) = 11.74$, $p < .001, \eta^2_G = .28$). Bonferroni-corrected post-hoc tests, performed separately within each target type, show significant differences between the Easy and Hard evolutions of the BB target (($t(11) = 4.26$, $p < 0.01$) and the HF target ($t(11) = 5.26$, $p < 0.01$).

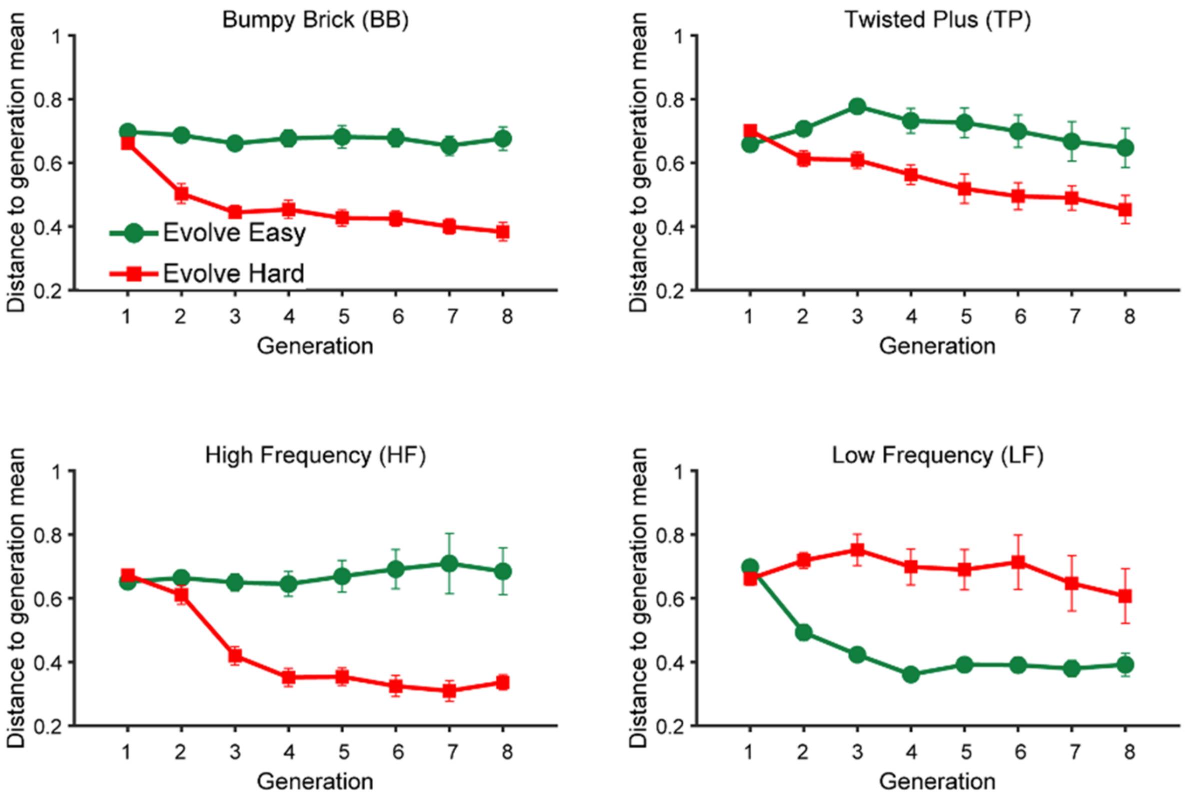

Figure B4. Average difference between individual distractors and the mean distractor in a given generation, measured as the L2 norm distance between their Fourier amplitude spectra, across generations for each target type and evolution condition. Error bars correspond to S.E.M.

Appendix C.

The first experiment showed that the genetic algorithm can successfully generate harder or easier distractors for a target shape in a visual search task. However, it is unclear whether eight generations of evolution are sufficient to find the hardest possible (or easiest possible) distractors. In a control experiment, we tested observers for 20 generations of search for the 'Bump Brick' (BB) target in the Evolve-Hard condition to determine whether evolution continues past generation eight. Additionally, the mutation rate was systematically varied across blocks to test whether a 2% mutation rate is optimal, or whether increasing the mutation rate (allowing more noise in the distractor evolution process) would allow us to find even more difficult distractors.

Methods

Data were collected from 12 new participants (eight females, four males, mean age 28.7 years). All observers had normal or corrected-to normal vision and passed the Ishihara test for color vision. All experimental procedures were approved by the Brigham and Women's Hospital Institutional Review Board, and all participants gave informed consent and were paid $10 an hour.

Apparatus and stimuli

This experiment was identical to Experiment 1 with the following exceptions. There was only one shape target (BB) and the evolution condition was always set to make search more difficult (Evolve-Hard). The study consisted of three blocks, each with a different mutation rate (2%, 4%, or 8%) with the order of blocks counterbalanced between observers. Each block consisted of 20 generations of 24 trials. The trial procedure and distractor evolution were identical to Experiment 1.

A few display parameters were modified. Random variation was added to the sizes of items to reduce any effects from orthogonal low-level features like size; i.e. it would not be interesting if the algorithm evolved small distractors. Each shape was scaled to fit within a square with height/width chosen randomly from a uniform distribution with a range 135-165 pixels. The radii of the shapes were truncated at 0, to remove internal loops formed by sections of contour with a negative radius, as seen in the LF target in Experiment 1.

Results and Discussion

Figure C1 shows average RT by generation for each set size in the 3 mutation rate

conditions. The RT data show variability from one generation to the next. We smoothed the data by splitting RTs into 5 epochs (generation 1-4, 5-8, 9-12, 13-16 and 17-20), and averaged the RTs from each span to get a better sense of true evolution over time. A 3 (mutation rate: 2%, 4%, and 8%)) x 5 (epoch: 1-5) repeated measures ANOVA as factors show a main effect of generation ($F(4,44) = 4.07$, $p = .007, \eta^2_G = .27$) and no main effect of mutation rate ($F(2,22) = 1.18$, $p = .33$). The interaction between mutation rate and generation did not reach significance ($F(8,88) = 1.47$, $p = .18$).

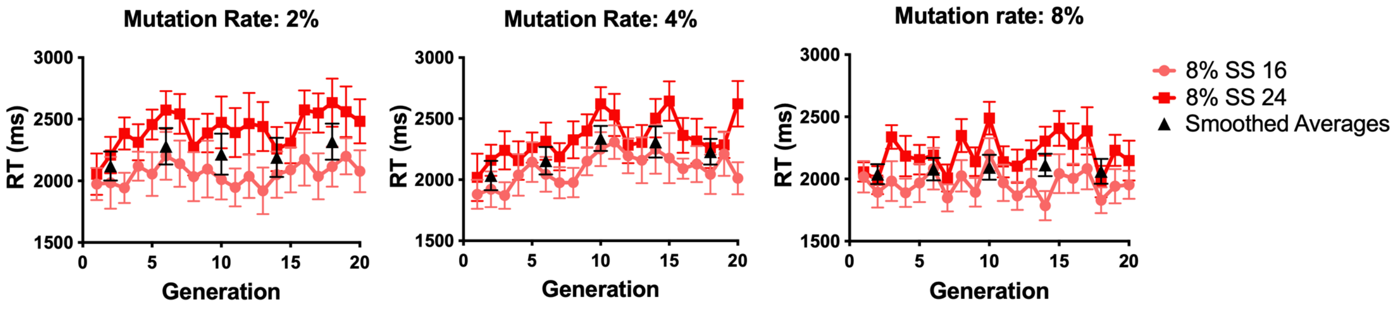

Figure C1. RT over generations for a mutation rate of 2,4, and 8%. Black triangles correspond to smoothed RT data over 5 epochs of 4 generations each. Error bars correspond to S.E.M. Notice the y-axis range is smaller than in Experiment 1 RT plots.

As there was no main effect of mutation rate, we collapsed over mutation rates to compare evolution over the 5 epochs. A Bonferroni correction for multiple comparison with 4 t-tests gives a threshold of $p < .0125$ ($p < .05/4$). The difference in RTs between epoch 1 (generations 1-4) and epoch 2 (generations 5-8) was significantly different $t(11) = 4.85$, $p<.001$, while the differences between subsequent epochs (2 versus 3, 3 versus 4, and 4 vs 5) were not significantly different (all $t<1.2$, all $p>.23$). This suggests the strongest evolution is taking place over generations 1-8 with performance remaining roughly asymptotic thereafter.

As in Experiment 1, we measure the similarity between distractors and the target using different shape parameterization methods. The results of this analysis are shown in Figure C2. For the shape skeleton target-distractor different measure used (from Ayzenberg & Lourenco (2019) a 3 (Mutation rate) x 5 (Epoch) ANOVA shows no main effect of epoch ($F(4,44) = 0.946$, $p = 0.45$) or mutation rate ($F(2,22) = 1.237$, $p = 0.31$) and no interaction ($F(8,88) = 1.737$, p =

0.10). For the perimeter^2/area target-distractor difference analysis, a 3 (Mutation rate) x 5 (Epoch) ANOVA shows no main effect of epoch (F(4,44) = 0.53, p = 0.714) or mutation rate (F(2,22) = 0.73, p = 0.49) and no interaction (F(8,88) = 0.44, p = 0.90). The radial frequency analysis, which calculated the target-distractor difference as the distance (L2 norm) between the radial Fourier amplitude spectra for each epoch. A 5 (epoch) x 3 (mutation rate) repeated measures ANOVA shows a main effect of epoch ($F(4,44) = 3.97, p < .01, \eta_G^2 = .033$) but no significant effect of mutation rate ($F(2,22) = .019, p = .98$) and no interaction ($F(8,88) = 0.77, p = .63$). Bonferroni-corrected t-tests between the individual epochs show significant differences between the first and second epoch ($t(11) = 5.02, p < 0.01$) and between the first and third epoch ($t(11) = 4.56, p < 0.01$); however, none of the other epochs differ from each other.

According to the skeleton and perimeter^2/area shape parameterization, the frequency manipulations halted distractor evolution – the distractors did not become any more or less similar to the target over generations. These target-distractor difference metrics for the skeleton and perimeter^2/area representations showed no evolution in experiment 1 as well. However, the interpretation that evolution is halted is inconsistent with the RT findings presented in Figure C1, which suggests the strongest distractor evolution must be taking place in earlier generations. The radial frequency analysis suggests that most of the change in distractor shape occurs during the first four or so generations and further evolution does not produce more difficult search – findings that are consistent with the RT results. The radial frequency target-distractor difference was most effective and aligned with the RT findings to suggests that after about eight generations, the algorithm has converged; meaning, it has found what it is going to find for a specific target and starting distractors. However, the convergence rate is dependent on the parameters of the genetic algorithm and it is possible that other implementations would behave differently.

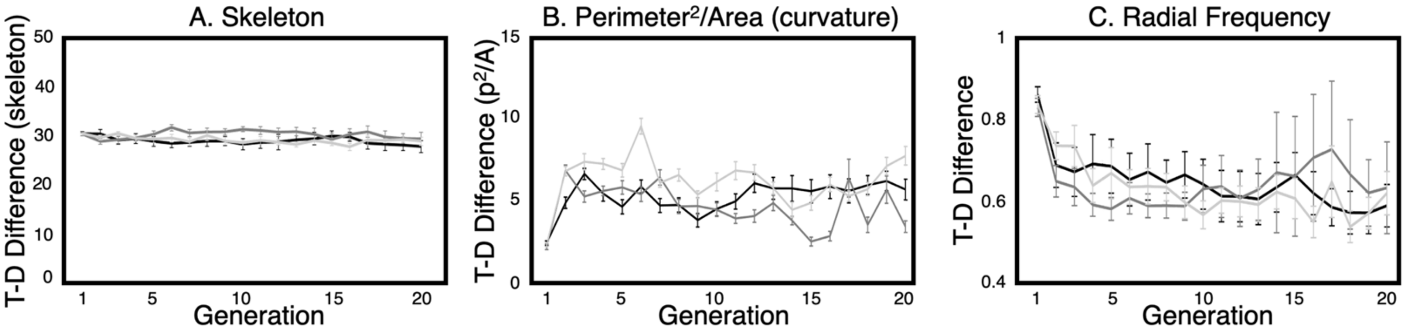

Figure *C2*. Average target-distractor difference for different shape parameters over generations for each mutation rate. Error bars correspond to S.E.M.

The target-distractor difference over generations for the three mutation rates from Experiment 2 is shown in Figure C2.

Appendix D.

As in the previous experiments, we measure the similarity between distractors and the target using different shape parameterization methods. The results for the skeleton representation are shown in figure D1, the results from experiment 1 are included for comparison. A 2 (evolution direction) x 4 (target type) ANOVA showed no main effect of evolution direction ($F(1,11) = 1.85$, $p = 0.20$) or target type ($F(3,33) = 0.840$, $p = 0.48$) and no interaction ($F(3,33) = 0.96$, $p = 0.43$). According to the skeleton distance measure, the frequency manipulations completely halted distractor evolution, an interpretation which is inconsistent with the RT findings.

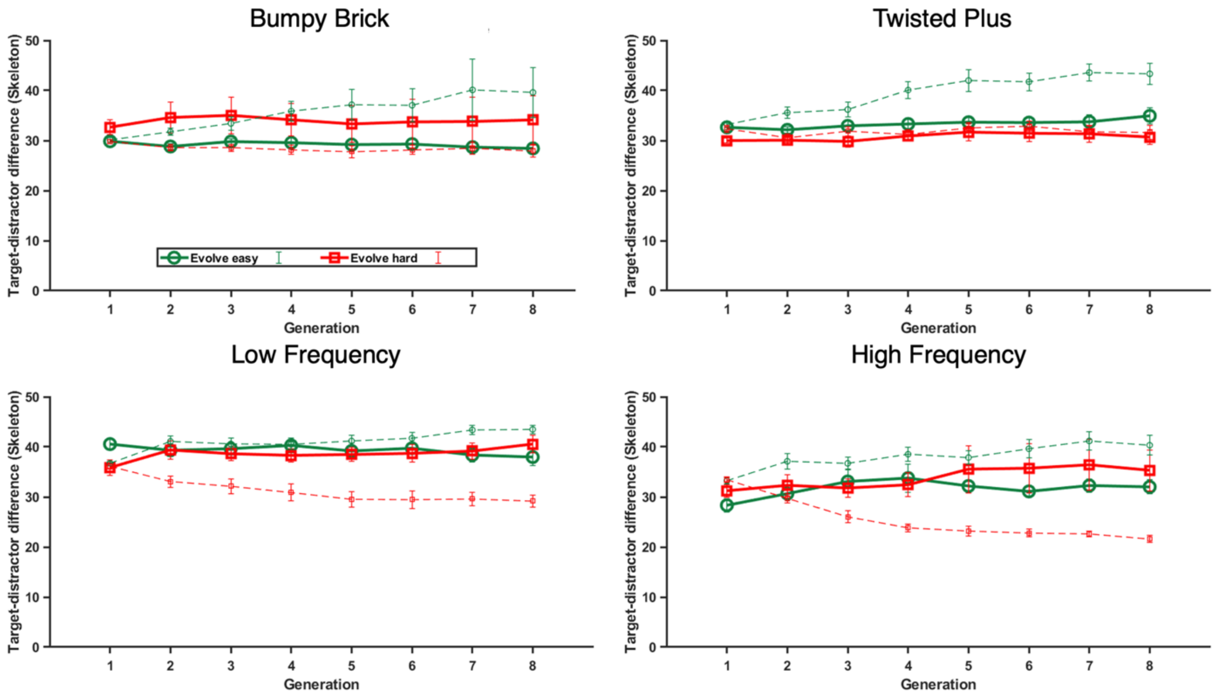

Figure D1. Average target-distractor difference (measured as the distance between target-distractor shape skeletons) over generations for each target type and evolution condition. Dotted line corresponds to data from experiment 1 and error bars correspond to S.E.M.

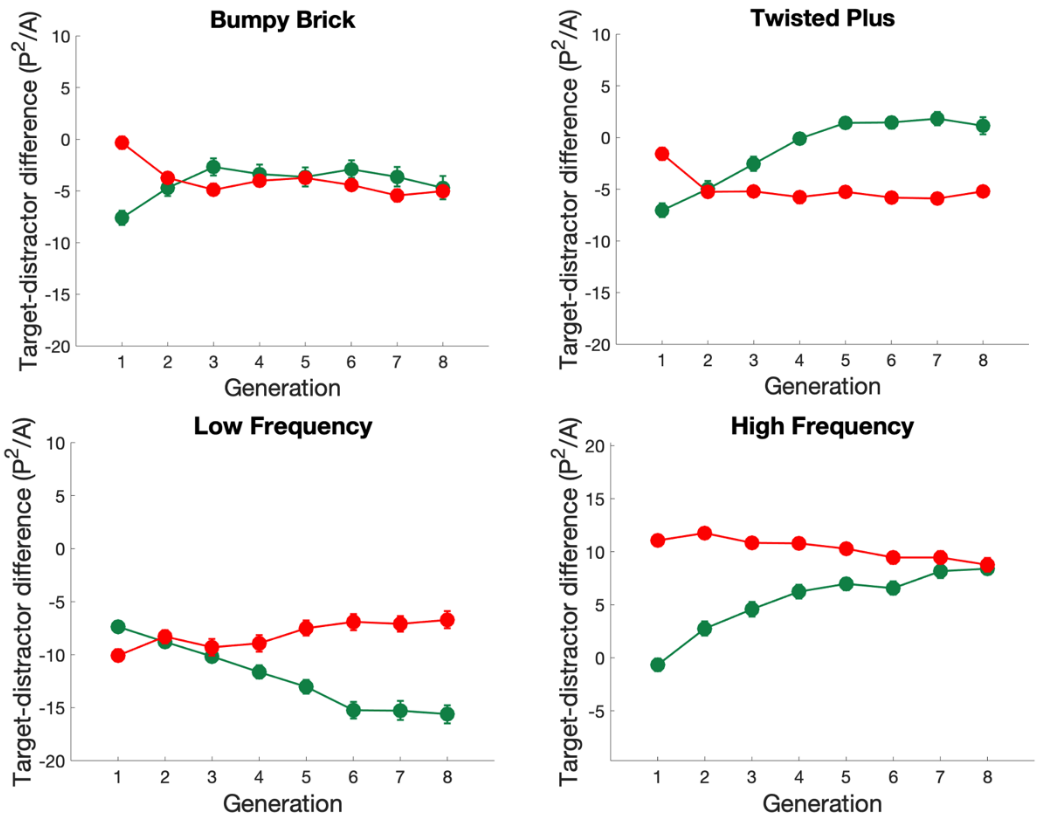

Figure D2. Average target-distractor difference (measured as the distance between target-distractor total curvature) over generations for each target type and evolution condition. Error bars correspond to S.E.M.

The results for the target-distractor distance for total curvature are shown in Figure D2. A 2 (evolution direction) x 4 (target type) ANOVA showed a main effect of evolution direction (F(1,11) = 9.99, *p* = 0.009) and target type (F(3,33) = 2.78, *p* = 0.06) and an interaction (F(3,33) = 0.37.11, *p* <0.001). Bonferroni-corrected t-tests between the difference in generation 1 and 8 for Evolve-Easy and Evolve-Hard show significant differences for all targets except the Bumpy Brick (p <.002, except Bumpy Brick p = .01, which does not reach significance with the Bonferroni correction). However, these target-distractor difference measures for total curvature fail to align with key characteristics in the RT performance. There was a general trend where evolution leads to an easier search but fails to make the low and high frequency targets harder to find. That pattern is not reflected in the evolve hard case for the Low Frequency target,

additionally, the RT changes documented in the Bumpy Brick and Twisted Plus condition are less robust than one might expect based on the RT results.

The measure of similarity for radial the target and distractor amplitude spectra is shown in Figure D3. Results for Experiment 1 are included for comparison. It is clear, as in the RT data, that the genetic algorithm is producing an evolutionary effect for two of the target types and is doing nothing for the other two. A 2 (evolution condition) x 4 (target type) repeated measures ANOVA of the change in target and distractor amplitude spectra between the first and last generation shows a main effect of evolution direction ($F(1,11) = 19.8$, $p < .001, \eta^2_G = .12$), no main effect of target type ($F(3,33) = 0.19$, $p = .90$), but a significant interaction ($F(3,33) = 10.8$, $p < .01, \eta^2_G = .15$). Bonferroni-corrected post-hoc tests show a significant difference between the Evolve-Easy and Evolve-Hard conditions for the TP target *(*$t(11) = 4.33$, $p < 0.01$) but no significant differences between these conditions for the Low Frequency and High Frequency targets. The BB results are suggestive but not statistically significant ($t(11) = 2.16$, $p = 0.054$). This suggests that, despite the restrictions on the algorithm, it is able to generate distractors that are more similar to the TP target in the Evolve-Hard condition and less similar in the Evolve-Easy condition. However, this was not possible for two of the other targets and may not have been possible for the BB target. The findings from the similarity analysis show that the RT findings are explained by radial frequency mechanisms, the skeleton and total curvature distance metrics failed to explain key patterns in the RT data.

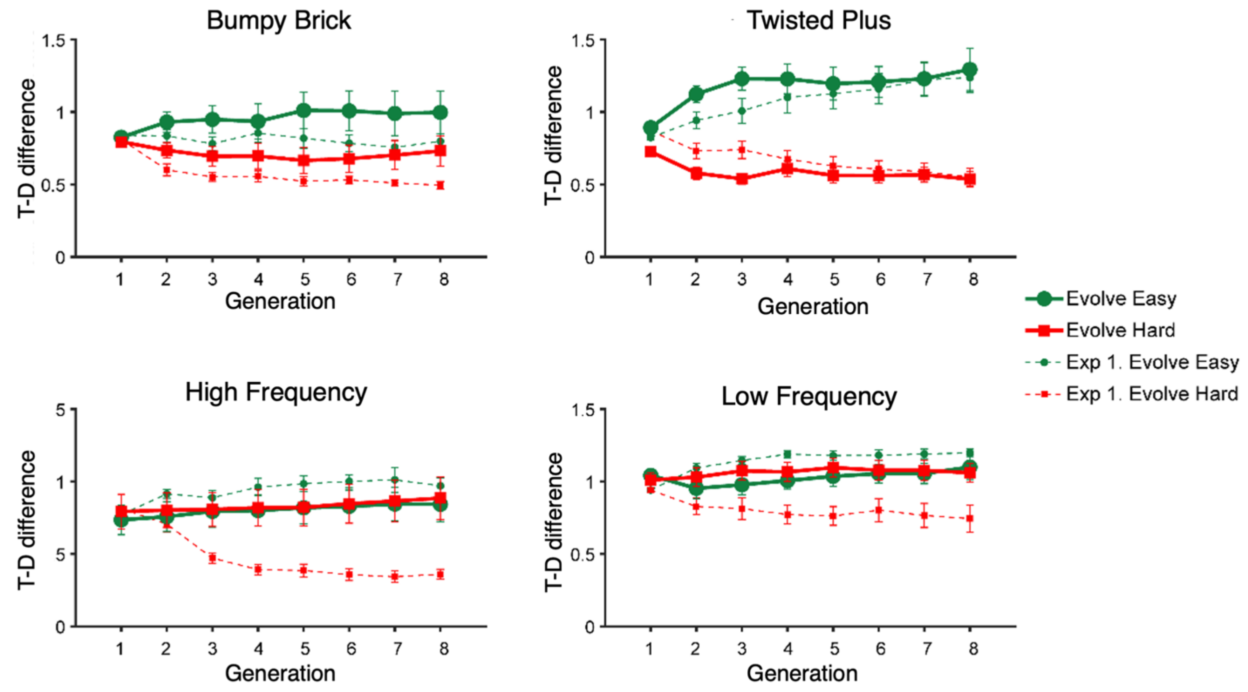

Figure D3. Average target-distractor difference (measured as the L2 norm distance between their Fourier amplitude spectra) over generations for each target type and evolution condition. Dotted line corresponds to data from experiment 1 and error bars correspond to S.E.M.

Appendix E.

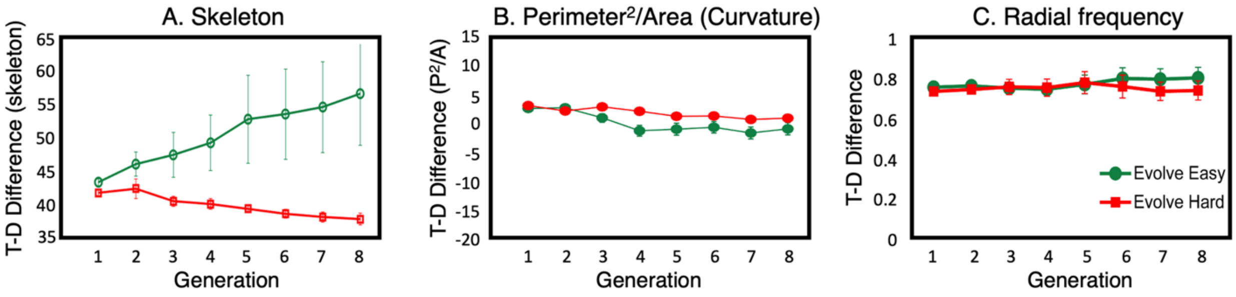

Figure E1. Average target-distractor difference for search set to Evolve-Hard or Evolve-Easy with a rabbit shaped silhouette as a target. Error bars correspond to S.E.M.

Figure E1 shows the similarity between the rabbit target and the distractor shapes over generations. For the skeleton target-distractor different measure used (from Ayzenberg & Lourenco (2019)) the Evolve-Hard and Evolve-Easy conditions are significantly different from each other at generation 8 ($t(12) = 2.498$, $p < 0.05$). In the Evolve-Hard condition, generation 8 is significantly different from generation 1 ($t(12) = 3.185$, $p < 0.01$) but in the Evolve-Easy condition, generation 8 is not significantly different from generation 1 ($t(12) = 1.690$, $p = 0.12$). The Fourier-amplitude spectra similarity measure and total curvature measure showed relatively little change over generations when considering target-distractor distance,. The change in similarity between the first and last generation is not significantly different in these two evolution conditions, both when considering Fourier-Amplitude ($t(12) = 0.6801$, $p = 0.5093$) and total curvature ($t(12) = -0.46$, $p = 0.54$). The target-distractor similarity is somewhat constrained in this experiment because the distractors were restricted to the first ten radial frequencies while the target included higher frequencies. However, restricting the analysis to just the ten lowest radial frequencies gives similar results.

According to the Target-distractor difference for perimeter^2/area and Fourier-amplitude shape parameterization, distractor evolution was halted with the rabbit target– the distractors did not become any more or less similar to the target over generations. However, the interpretation that evolution is halted is inconsistent with the RT changes over generation (in Figure 14) . The skeleton distance measure best captured target-distractor changes consistent with RT behavior over generations in the Evolve-Hard case.
